# Integrated global assessment of the natural forest carbon potential

**DOI:** 10.1038/s41586-023-06723-z

**Published:** 2023-11-13

**Authors:** Lidong Mo, Constantin M. Zohner, Peter B. Reich, Jingjing Liang, Sergio de Miguel, Gert-Jan Nabuurs, Susanne S. Renner, Johan van den Hoogen, Arnan Araza, Martin Herold, Leila Mirzagholi, Haozhi Ma, Colin Averill, Oliver L. Phillips, Javier G. P. Gamarra, Iris Hordijk, Devin Routh, Meinrad Abegg, Yves C. Adou Yao, Giorgio Alberti, Angelica M. Almeyda Zambrano, Braulio Vilchez Alvarado, Esteban Alvarez-Dávila, Patricia Alvarez-Loayza, Luciana F. Alves, Iêda Amaral, Christian Ammer, Clara Antón-Fernández, Alejandro Araujo-Murakami, Luzmila Arroyo, Valerio Avitabile, Gerardo A. Aymard, Timothy R. Baker, Radomir Bałazy, Olaf Banki, Jorcely G. Barroso, Meredith L. Bastian, Jean-Francois Bastin, Luca Birigazzi, Philippe Birnbaum, Robert Bitariho, Pascal Boeckx, Frans Bongers, Olivier Bouriaud, Pedro H. S. Brancalion, Susanne Brandl, Francis Q. Brearley, Roel Brienen, Eben N. Broadbent, Helge Bruelheide, Filippo Bussotti, Roberto Cazzolla Gatti, Ricardo G. César, Goran Cesljar, Robin L. Chazdon, Han Y. H. Chen, Chelsea Chisholm, Hyunkook Cho, Emil Cienciala, Connie Clark, David Clark, Gabriel D. Colletta, David A. Coomes, Fernando Cornejo Valverde, José J. Corral-Rivas, Philip M. Crim, Jonathan R. Cumming, Selvadurai Dayanandan, André L. de Gasper, Mathieu Decuyper, Géraldine Derroire, Ben DeVries, Ilija Djordjevic, Jiri Dolezal, Aurélie Dourdain, Nestor Laurier Engone Obiang, Brian J. Enquist, Teresa J. Eyre, Adandé Belarmain Fandohan, Tom M. Fayle, Ted R. Feldpausch, Leandro V. Ferreira, Leena Finér, Markus Fischer, Christine Fletcher, Lorenzo Frizzera, Damiano Gianelle, Henry B. Glick, David J. Harris, Andrew Hector, Andreas Hemp, Geerten Hengeveld, Bruno Hérault, John L. Herbohn, Annika Hillers, Eurídice N. Honorio Coronado, Cang Hui, Thomas Ibanez, Nobuo Imai, Andrzej M. Jagodziński, Bogdan Jaroszewicz, Vivian Kvist Johannsen, Carlos A. Joly, Tommaso Jucker, Ilbin Jung, Viktor Karminov, Kuswata Kartawinata, Elizabeth Kearsley, David Kenfack, Deborah K. Kennard, Sebastian Kepfer-Rojas, Gunnar Keppel, Mohammed Latif Khan, Timothy J. Killeen, Hyun Seok Kim, Kanehiro Kitayama, Michael Köhl, Henn Korjus, Florian Kraxner, Dmitry Kucher, Diana Laarmann, Mait Lang, Huicui Lu, Natalia V. Lukina, Brian S. Maitner, Yadvinder Malhi, Eric Marcon, Beatriz Schwantes Marimon, Ben Hur Marimon-Junior, Andrew R. Marshall, Emanuel H. Martin, Jorge A. Meave, Omar Melo-Cruz, Casimiro Mendoza, Irina Mendoza-Polo, Stanislaw Miscicki, Cory Merow, Abel Monteagudo Mendoza, Vanessa S. Moreno, Sharif A. Mukul, Philip Mundhenk, María Guadalupe Nava-Miranda, David Neill, Victor J. Neldner, Radovan V. Nevenic, Michael R. Ngugi, Pascal A. Niklaus, Jacek Oleksyn, Petr Ontikov, Edgar Ortiz-Malavasi, Yude Pan, Alain Paquette, Alexander Parada-Gutierrez, Elena I. Parfenova, Minjee Park, Marc Parren, Narayanaswamy Parthasarathy, Pablo L. Peri, Sebastian Pfautsch, Nicolas Picard, Maria Teresa F. Piedade, Daniel Piotto, Nigel C. A. Pitman, Axel Dalberg Poulsen, John R. Poulsen, Hans Pretzsch, Freddy Ramirez Arevalo, Zorayda Restrepo-Correa, Mirco Rodeghiero, Samir G. Rolim, Anand Roopsind, Francesco Rovero, Ervan Rutishauser, Purabi Saikia, Christian Salas-Eljatib, Philippe Saner, Peter Schall, Mart-Jan Schelhaas, Dmitry Schepaschenko, Michael Scherer-Lorenzen, Bernhard Schmid, Jochen Schöngart, Eric B. Searle, Vladimír Seben, Josep M. Serra-Diaz, Douglas Sheil, Anatoly Z. Shvidenko, Javier E. Silva-Espejo, Marcos Silveira, James Singh, Plinio Sist, Ferry Slik, Bonaventure Sonké, Alexandre F. Souza, Krzysztof J. Stereńczak, Jens-Christian Svenning, Miroslav Svoboda, Ben Swanepoel, Natalia Targhetta, Nadja Tchebakova, Hans ter Steege, Raquel Thomas, Elena Tikhonova, Peter M. Umunay, Vladimir A. Usoltsev, Renato Valencia, Fernando Valladares, Fons van der Plas, Tran Van Do, Michael E. van Nuland, Rodolfo M. Vasquez, Hans Verbeeck, Helder Viana, Alexander C. Vibrans, Simone Vieira, Klaus von Gadow, Hua-Feng Wang, James V. Watson, Gijsbert D. A. Werner, Susan K. Wiser, Florian Wittmann, Hannsjoerg Woell, Verginia Wortel, Roderik Zagt, Tomasz Zawiła-Niedźwiecki, Chunyu Zhang, Xiuhai Zhao, Mo Zhou, Zhi-Xin Zhu, Irie C. Zo-Bi, George D. Gann, Thomas W. Crowther

**Affiliations:** 1 Institute of Integrative Biology, ETH Zurich (Swiss Federal Institute of Technology), Zurich, Switzerland; 2 Department of Forest Resources, University of Minnesota, St. Paul, MN USA; 3 Hawkesbury Institute for the Environment, Western Sydney University, Penrith, New South Wales Australia; 4 Institute for Global Change Biology, University of Michigan, Ann Arbor, MI USA; 5 Department of Forestry and Natural Resources, Purdue University, West Lafayette, IN USA; 6 Department of Agricultural and Forest Sciences and Engineering, University of Lleida, Lleida, Spain; 7 Joint Research Unit CTFC - AGROTECNIO - CERCA, Solsona, Spain; 8 Wageningen University & Research, Wageningen, The Netherlands; 9 Department of Biology, Washington University, St. Louis, MO USA; 10 Remote Sensing and Geoinformatics Section, Helmholtz GFZ German Research Centre for Geosciences, Potsdam, Germany; 11 School of Geography, University of Leeds, Leeds, UK; 12 Forestry Division, Food and Agriculture Organization of the United Nations, Rome, Italy; 13 Central IT - Teaching and Research, University of Zürich, Zürich, Switzerland; 14 Swiss Federal Institute for Forest, Snow and Landscape Research WSL, Birmensdorf, Switzerland; 15 UFR Biosciences, University Félix Houphouët-Boigny, Abidjan, Côte d’Ivoire; 16 Department of Agricultural, Food, Environmental and Animal Sciences, University of Udine, Udine, Italy; 17 National Biodiversity Future Center (NBFC), Palermo, Italy; 18 Spatial Ecology and Conservation Lab, Center for Latin American Studies, University of Florida, Gainesville, FL USA; 19 Forestry School, Tecnológico de Costa Rica TEC, Cartago, Costa Rica; 20 Fundacion ConVida, Universidad Nacional Abierta y a Distancia, UNAD, Medellín, Colombia; 21 Field Museum of Natural History, Chicago, IL USA; 22 Center for Tropical Research, Institute of the Environment and Sustainability, University of California, Los Angeles (UCLA), Los Angeles, CA USA; 23 National Institute of Amazonian Research, Manaus, Brazil; 24 Silviculture and Forest Ecology of the Temperate Zones, University of Göttingen, Göttingen, Germany; 25 Division of Forest and Forest Resources, Norwegian Institute of Bioeconomy Research (NIBIO), Ås, Norway; 26 Museo de Historia Natural Noel Kempff Mercado, Santa Cruz de la Sierra, Bolivia; 27 Joint Research Centre, European Commission, Ispra, Italy; 28 Programa de Ciencias del Agro y el Mar, Herbario Universitario (PORT), UNELLEZ-Guanare, Portuguesa, Venezuela; 29 Compensation International Progress S. A. Ciprogress Greenlife, Bogotá, Colombia; 30 Department of Geomatics, Forest Research Institute, Sękocin Stary, Poland; 31 Naturalis Biodiversity Center, Leiden, The Netherlands; 32 Centro Multidisciplinar, Universidade Federal do Acre, Rio Branco, Brazil; 33 Proceedings of the National Academy of Sciences, Washington, DC USA; 34 Department of Evolutionary Anthropology, Duke University, Durham, NC USA; 35 TERRA Teaching and Research Centre, Gembloux Agro-Bio Tech, University of Liege, Liege, Belgium; 36 Forestry Consultant, Grosseto, Italy; 37 Institut Agronomique néo-Calédonien (IAC), Nouméa, New Caledonia; 38 AMAP, Univ. Montpellier, Montpellier, France; 39 CIRAD, CNRS, INRAE, IRD, Montpellier, France; 40 Institute of Tropical Forest Conservation, Mbarara University of Science & Technology, Mbarara, Uganda; 41 Isotope Bioscience Laboratory - ISOFYS, Ghent University, Ghent, Belgium; 42 Ștefan cel Mare University of Suceava, Suceava, Romania; 43 Department of Forest Sciences, Luiz de Queiroz College of Agriculture, University of São Paulo, Piracicaba, Brazil; 44 Bavarian State Institute of Forestry, Freising, Germany; 45 Department of Natural Sciences, Manchester Metropolitan University, Manchester, UK; 46 Institute of Biology/Geobotany and Botanical Garden, Martin Luther University Halle-Wittenberg, Halle-Wittenberg, Germany; 47 German Centre for Integrative Biodiversity Research (iDiv) Halle-Jena-Leipzig, Leipzig, Germany; 48 Department of Agriculture, Food, Environment and Forest (DAGRI), University of Firenze, Florence, Italy; 49 Department of Biological, Geological, and Environmental Sciences, University of Bologna, Bologna, Italy; 50 Department of Spatial Regulation, GIS and Forest Policy, Institute of Forestry, Belgrade, Serbia; 51 Department of Ecology and Evolutionary Biology, University of Connecticut, Storrs, CT USA; 52 Tropical Forests and People Research Centre, University of the Sunshine Coast, Sippy Downs, Queensland Australia; 53 Faculty of Natural Resources Management, Lakehead University, Thunder Bay, Ontario Canada; 54 Division of Forest Resources Information, Korea Forest Promotion Institute, Seoul, South Korea; 55 IFER - Institute of Forest Ecosystem Research, Jilove u Prahy, Czech Republic; 56 Global Change Research Institute CAS, Brno, Czech Republic; 57 Nicholas School of the Environment, Duke University, Durham, NC USA; 58 Department of Biology, University of Missouri–St. Louis, St. Louis, MO USA; 59 Programa de Pós-graduação em Biologia Vegetal, Instituto de Biologia, Universidade Estadual de Campinas, Campinas, Brazil; 60 Conservation Research Institute, Department of Plant Sciences, University of Cambridge, Cambridge, UK; 61 Andes to Amazon Biodiversity Program, Madre de Dios, Peru; 62 Facultad de Ciencias Forestales y Ambientales, Universidad Juárez del Estado de Durango, Durango, Mexico; 63 Department of Biology, West Virginia University, Morgantown, WV USA; 64 Department of Physical and Biological Sciences, The College of Saint Rose, Albany, NY USA; 65 Biology Department, Centre for Structural and Functional Genomics, Concordia University, Montreal, Quebec Canada; 66 Natural Science Department, Universidade Regional de Blumenau, Blumenau, Brazil; 67 Cirad, UMR EcoFoG (AgroParisTech, CNRS, INRAE, Université des Antilles, Université de la Guyane), Campus Agronomique, Kourou, French Guiana; 68 Department of Geography, Environment and Geomatics, University of Guelph, Guelph, Ontario Canada; 69 Institute of Forestry, Belgrade, Serbia; 70 Institute of Botany, The Czech Academy of Sciences, Třeboň, Czech Republic; 71 Department of Botany, Faculty of Science, University of South Bohemia, České Budějovice, Czech Republic; 72 IRET, Herbier National du Gabon (CENAREST), Libreville, Gabon; 73 Department of Ecology and Evolutionary Biology, University of Arizona, Tucson, AZ USA; 74 The Santa Fe Institute, Santa Fe, NM USA; 75 Department of Environment and Science, Queensland Herbarium and Biodiversity Science, Toowong, Queensland Australia; 76 Ecole de Foresterie et Ingénierie du Bois, Université Nationale d’Agriculture, Kétou, Benin; 77 School of Biological and Behavioural Sciences, Queen Mary University of London, London, UK; 78 Biology Centre of the Czech Academy of Sciences, Institute of Entomology, České Budějovice, Czech Republic; 79 Geography, Faculty of Environment, Science and Economy, University of Exeter, Exeter, UK; 80 Museu Paraense Emílio Goeldi, Coordenação de Ciências da Terra e Ecologia, Belém, Brazil; 81 Natural Resources Institute Finland (Luke), Joensuu, Finland; 82 Institute of Plant Sciences, University of Bern, Bern, Switzerland; 83 Forest Research Institute Malaysia, Kuala Lumpur, Malaysia; 84 Research and Innovation Centre, Fondazione Edmund Mach, San Michele All’adige, Italy; 85 Glick Designs LLC, Hadley, MA USA; 86 Royal Botanic Garden Edinburgh, Edinburgh, UK; 87 Department of Biology, University of Oxford, Oxford, UK; 88 Department of Plant Systematics, University of Bayreuth, Bayreuth, Germany; 89 Cirad, UPR Forêts et Sociétés, University of Montpellier, Montpellier, France; 90 Department of Forestry and Environment, National Polytechnic Institute (INP-HB), Yamoussoukro, Côte d’Ivoire; 91 Forest Research Institute, University of the Sunshine Coast, Sippy Downs, Queensland Australia; 92 Centre for Conservation Science, The Royal Society for the Protection of Birds, Sandy, UK; 93 Wild Chimpanzee Foundation, Liberia Office, Monrovia, Liberia; 94 Instituto de Investigaciones de la Amazonía Peruana, Iquitos, Peru; 95 Centre for Invasion Biology, Department of Mathematical Sciences, Stellenbosch University, Stellenbosch, South Africa; 96 Theoretical Ecology Unit, African Institute for Mathematical Sciences, Cape Town, South Africa; 97 AMAP, Univ. Montpellier, CIRAD, CNRS, INRAE, IRD, Montpellier, France; 98 Department of Forest Science, Tokyo University of Agriculture, Tokyo, Japan; 99 Institute of Dendrology, Polish Academy of Sciences, Kórnik, Poland; 100 Department of Game Management and Forest Protection, Poznań University of Life Sciences, Poznań, Poland; 101 Faculty of Biology, Białowieża Geobotanical Station, University of Warsaw, Białowieża, Poland; 102 Department of Geosciences and Natural Resource Management, University of Copenhagen, Copenhagen, Denmark; 103 Department of Plant Biology, Institute of Biology, University of Campinas, UNICAMP, Campinas, Brazil; 104 School of Biological Sciences, University of Bristol, Bristol, UK; 105 Forestry Faculty, Mytischi Branch of Bauman Moscow State Technical University, Mytischi, Russian Federation; 106 Negaunee Integrative Research Center, Field Museum of Natural History, Chicago, IL USA; 107 CAVElab - Computational & Applied Vegetation Ecology, Department of Environment, Ghent University, Ghent, Belgium; 108 CTFS-ForestGEO, Smithsonian Tropical Research Institute, Balboa, Panama; 109 Department of Physical and Environmental Sciences, Colorado Mesa University, Grand Junction, CO USA; 110 UniSA STEM and Future Industries Institute, University of South Australia, Adelaide, South Australia Australia; 111 Department of Botany, Dr. Harisingh Gour Vishwavidyalaya (A Central University), Sagar, India; 112 Department of Agriculture, Forestry and Bioresources, Seoul National University, Seoul, South Korea; 113 Interdisciplinary Program in Agricultural and Forest Meteorology, Seoul National University, Seoul, South Korea; 114 National Center for Agro Meteorology, Seoul, South Korea; 115 Research Institute for Agriculture and Life Sciences, Seoul National University, Seoul, South Korea; 116 Graduate School of Agriculture, Kyoto University, Kyoto, Japan; 117 Institute for World Forestry, University of Hamburg, Hamburg, Germany; 118 Institute of Forestry and Engineering, Estonian University of Life Sciences, Tartu, Estonia; 119 Biodiversity and Natural Resources Program, International Institute for Applied Systems Analysis, Laxenburg, Austria; 120 Peoples’ Friendship University of Russia (RUDN University), Moscow, Russian Federation; 121 Faculty of Forestry, Qingdao Agricultural University, Qingdao, China; 122 Center for Forest Ecology and Productivity, Russian Academy of Sciences, Moscow, Russian Federation; 123 Environmental Change Institute, School of Geography and the Environment, University of Oxford, Oxford, UK; 124 AgroParisTech, UMR-AMAP, Cirad, CNRS, INRA, IRD, Université de Montpellier, Montpellier, France; 125 Departamento de Ciências Biológicas, Universidade do Estado de Mato Grosso, Nova Xavantina, Brazil; 126 Department of Environment and Geography, University of York, York, UK; 127 Flamingo Land Ltd., Kirby Misperton, UK; 128 Department of Wildlife Management, College of African Wildlife Management, Mweka, Tanzania; 129 Departamento de Ecología y Recursos Naturales, Facultad de Ciencias, Universidad Nacional Autónoma de México, Mexico City, Mexico; 130 Universidad del Tolima, Ibagué, Colombia; 131 Colegio de Profesionales Forestales de Cochabamba, Cochabamba, Bolivia; 132 Jardín Botánico de Medellín, Medellín, Colombia; 133 Department of Forest Management, Dendrometry and Forest Economics, Warsaw University of Life Sciences, Warsaw, Poland; 134 Jardín Botánico de Missouri, Oxapampa, Peru; 135 Universidad Nacional de San Antonio Abad del Cusco, Cusco, Peru; 136 Department of Environment and Development Studies, United International University, Dhaka, Bangladesh; 137 Instituto de Silvicultura e Industria de la Madera, Universidad Juárez del Estado de Durango, Durango, Mexico; 138 Programa de Doctorado en Ingeniería para el Desarrollo Rural y Civil, Escuela de Doctorado Internacional de la Universidad de Santiago de Compostela (EDIUS), Santiago de Compostela, Spain; 139 Universidad Estatal Amazónica, Puyo, Ecuador; 140 Department of Evolutionary Biology and Environmental Studies, University of Zürich, Zürich, Switzerland; 141 Climate, Fire, and Carbon Cycle Sciences, USDA Forest Service, Durham, NH USA; 142 Centre for Forest Research, Université du Québec à Montréal, Montréal, Quebec Canada; 143 V. N. Sukachev Institute of Forest, FRC KSC, Siberian Branch of the Russian Academy of Sciences, Krasnoyarsk, Russian Federation; 144 Forest Ecology and Forest Management Group, Wageningen University & Research, Wageningen, The Netherlands; 145 Department of Ecology and Environmental Sciences, Pondicherry University, Puducherry, India; 146 Instituto Nacional de Tecnología Agropecuaria (INTA), Universidad Nacional de la Patagonia Austral (UNPA), Consejo Nacional de Investigaciones Científicas y Técnicas (CONICET), Río Gallegos, Argentina; 147 School of Social Sciences (Urban Studies), Western Sydney University, Penrith, New South Wales Australia; 148 GIP Ecofor, Paris, France; 149 Instituto Nacional de Pesquisas da Amazônia, Manaus, Brazil; 150 Laboratório de Dendrologia e Silvicultura Tropical, Centro de Formação em Ciências Agroflorestais, Universidade Federal do Sul da Bahia, Itabuna, Brazil; 151 The Nature Conservancy, Boulder, CO USA; 152 Chair of Forest Growth and Yield Science, Department of Life Science Systems, TUM School of Life Sciences, Technical University of Munich, Freising, Germany; 153 Sustainable Forest Management Research Institute (iuFOR), University Valladolid, Valladolid, Spain; 154 Universidad Nacional de la Amazonía Peruana, Iquitos, Peru; 155 Servicios Ecosistémicos y Cambio Climático (SECC), Fundación Con Vida & Corporación COL-TREE, Medellín, Colombia; 156 Centro Agricoltura, Alimenti, Ambiente, University of Trento, San Michele All’adige, Italy; 157 Center for Natural Climate Solutions, Conservation International, Arlington, VA USA; 158 Department of Biology, University of Florence, Florence, Italy; 159 Tropical Biodiversity Section, MUSE - Museo delle Scienze, Trento, Italy; 160 Info Flora, Geneva, Switzerland; 161 Department of Environmental Sciences, Central University of Jharkhand, Ranchi, India; 162 Vicerrectoría de Investigación y Postgrado, Universidad de La Frontera, Temuco, Chile; 163 Departamento de Gestión Forestal y su Medio Ambiente, Universidad de Chile, Santiago, Chile; 164 Rhino and Forest Fund e.V., Kehl, Germany; 165 Siberian Federal University, Krasnoyarsk, Russian Federation; 166 Geobotany, Faculty of Biology, University of Freiburg, Freiburg im Breisgau, Germany; 167 Remote Sensing Laboratories, Department of Geography, University of Zürich, Zürich, Switzerland; 168 National Forest Centre, Forest Research Institute Zvolen, Zvolen, Slovakia; 169 Université de Lorraine, AgroParisTech, INRAE, Silva, Nancy, France; 170 Center for Biodiversity Dynamics in a Changing World (BIOCHANGE), Department of Biology, Aarhus University, Aarhus, Denmark; 171 Faculty of Environmental Sciences and Natural Resource Management, Norwegian University of Life Sciences, Ås, Norway; 172 Departamento de Biología, Universidad de la Serena, La Serena, Chile; 173 Centro de Ciências Biológicas e da Natureza, Universidade Federal do Acre, Rio Branco, Brazil; 174 Guyana Forestry Commission, Georgetown, French Guiana; 175 Environmental and Life Sciences, Faculty of Science, Universiti Brunei Darussalam, Gadong, Brunei Darussalam; 176 Plant Systematic and Ecology Laboratory, Department of Biology, Higher Teachers’ Training College, University of Yaoundé I, Yaoundé, Cameroon; 177 Departamento de Ecologia, Universidade Federal do Rio Grande do Norte, Natal, Brazil; 178 Center for Ecological Dynamics in a Novel Biosphere (ECONOVO) & Center for Biodiversity Dynamics in a Changing World (BIOCHANGE), Department of Biology, Aarhus University, Aarhus, Denmark; 179 Section for Ecoinformatics and Biodiversity, Department of Biology, Aarhus University, Aarhus, Denmark; 180 Faculty of Forestry and Wood Sciences, Czech University of Life Sciences, Prague, Czech Republic; 181 Wildlife Conservation Society, New York, NY USA; 182 Quantitative Biodiversity Dynamics, Department of Biology, Utrecht University, Utrecht, The Netherlands; 183 Iwokrama International Centre for Rainforest Conservation and Development (IIC), Georgetown, French Guiana; 184 School of Forestry and Environmental Studies, Yale University, New Haven, CT USA; 185 Botanical Garden of Ural Branch of Russian Academy of Sciences, Ural State Forest Engineering University, Yekaterinburg, Russian Federation; 186 Pontificia Universidad Católica del Ecuador, Quito, Ecuador; 187 LINCGlobal, Museo Nacional de Ciencias Naturales, CSIC, Madrid, Spain; 188 Plant Ecology and Nature Conservation Group, Wageningen University, Wageningen, The Netherlands; 189 Silviculture Research Institute, Vietnamese Academy of Forest Sciences, Hanoi, Vietnam; 190 Department of Biology, Stanford University, Stanford, CA USA; 191 Centre for the Research and Technology of Agro-Environmental and Biological Sciences, CITAB, UTAD, Quinta de Prados, Vila Real, Portugal; 192 Agricultural High School, Polytechnic Institute of Viseu, IPV, Viseu, Portugal; 193 Department of Forest Engineering, Universidade Regional de Blumenau, Blumenau, Brazil; 194 Environmental Studies and Research Center, University of Campinas, UNICAMP, Campinas, Brazil; 195 Department of Forest and Wood Science, Stellenbosch University, Stellenbosch, South Africa; 196 Key Laboratory of Tropical Biological Resources, Ministry of Education, School of Life and Pharmaceutical Sciences, Hainan University, Haikou, China; 197 Division of Forestry and Natural Resources, West Virginia University, Morgantown, WV USA; 198 Department of Zoology, University of Oxford, Oxford, UK; 199 Manaaki Whenua – Landcare Research, Lincoln, New Zealand; 200 Department of Wetland Ecology, Institute of Geography and Geoecology, Karlsruhe Institute for Technology, Karlsruhe, Germany; 201 Independent Researcher, Bad Aussee, Austria; 202 Centre for Agricultural Research in Suriname (CELOS), Paramaribo, Suriname; 203 Tropenbos International, Wageningen, The Netherlands; 204 Polish State Forests, Coordination Center for Environmental Projects, Warsaw, Poland; 205 Research Center of Forest Management Engineering of State Forestry and Grassland Administration, Beijing Forestry University, Beijing, China; 206 Society for Ecological Restoration (SER), Washington, DC USA

**Keywords:** Forest ecology, Climate-change mitigation, Restoration ecology

## Abstract

Forests are a substantial terrestrial carbon sink, but anthropogenic changes in land use and climate have considerably reduced the scale of this system^1^. Remote-sensing estimates to quantify carbon losses from global forests^2–5^ are characterized by considerable uncertainty and we lack a comprehensive ground-sourced evaluation to benchmark these estimates. Here we combine several ground-sourced^6^ and satellite-derived approaches^2,7,8^ to evaluate the scale of the global forest carbon potential outside agricultural and urban lands. Despite regional variation, the predictions demonstrated remarkable consistency at a global scale, with only a 12% difference between the ground-sourced and satellite-derived estimates. At present, global forest carbon storage is markedly under the natural potential, with a total deficit of 226 Gt (model range = 151–363 Gt) in areas with low human footprint. Most (61%, 139 Gt C) of this potential is in areas with existing forests, in which ecosystem protection can allow forests to recover to maturity. The remaining 39% (87 Gt C) of potential lies in regions in which forests have been removed or fragmented. Although forests cannot be a substitute for emissions reductions, our results support the idea^2,3,9^ that the conservation, restoration and sustainable management of diverse forests offer valuable contributions to meeting global climate and biodiversity targets.

## Abstract (web summary)

Analysis of ground-sourced and satellite-derived models reveals a global forest carbon potential of 226 Gt outside agricultural and urban lands, with a difference of only 12% across these modelling approaches.

## Main

The continuing climate and biodiversity crises threaten ecosystems and human society^10,11^. Representing 80–90% of the global plant biomass^1^ and much of Earth’s terrestrial biodiversity^12^, forests play a key role in both climate-change mitigation and adaptation. So far, humans have removed almost half of Earth’s natural forests^13,14^, and we continue to lose a further 0.9–2.3 Gt C each year (about 15% of annual human carbon emissions) through deforestation^15^. In response to these pressing challenges, international environmental initiatives such as the UN Decade on Ecosystem Restoration^16^, the Kunming-Montreal Global Biodiversity Framework^17^ and the Glasgow Leaders’ Declaration on Forests and Land Use^18^ have been established to reduce deforestation and revitalize ecosystems. A key step in guiding such environmental targets is gaining a comprehensive understanding of the global distribution of existing forest carbon stocks, as well as the potential for carbon recapture if healthy ecosystems are allowed to recover^3,19^.

Remote-sensing observations have been central to the development of spatially continuous models of global forest biomass^2,7,8^. Building on these satellite-derived observations, a growing body of research has begun to use statistical extrapolations to estimate the potential extent of forest carbon stocks under natural conditions^2–4^. In recent years, refs. ^3,4^ combined remote-sensing forest-area estimates with coarse (ecoregion-level or country-level) carbon-storage estimates to approximate the global carbon potential. More recently, Walker et al.^2^ used satellite-derived biomass estimates from natural forested regions to statistically extrapolate potential forest biomass in the absence of human disturbance. Despite yielding carbon potential estimates ranging from 200 to 300 Gt C, inherent strengths and weaknesses of each approach have given rise to uncertainty across studies, with suggestions that these estimates may be up to 4–5 times too high^5,9,20,21^. As a result, confidence in the carbon potential of forest ecosystems remains low. Without an independent, bottom-up assessment of global forest carbon potential built directly from ground-sourced data, evaluating and benchmarking these satellite-derived trends remains challenging. Overcoming this controversy requires consideration of various independent approaches to identify the extent of confidence and uncertainty across different land uses around the world.

Another key challenge in the development of potential biomass estimates is how to approximate the ‘natural’ state of vegetation stocks. To do this, recent extrapolations of forest potential have been built from data collected in protected land^3^ or areas with minimal human disturbance^2^. However, a limitation of such approaches is that the focus on undisturbed areas restricts data to a few regions, which can bias results towards environments systematically avoided by humans. Protected areas may, for example, often exist in regions of marginal agricultural value or that possess unique ecological features^22^. An alternative approach to avoid such biases is to use observations across the full gradient of human disturbance and then use statistical techniques to remove the human footprint^23^. This method has proved successful in assessing the impact of historical human land use on soil carbon storage^23^. By allowing the inclusion of larger datasets across a broader range of environmental conditions, this approach has the potential to improve the statistical strength of biomass potential estimates. Consideration of the results from these different modelling datasets and approaches will be necessary to develop a comprehensive understanding of the global forest carbon potential.

Here we used a combination of independent modelling approaches to generate spatially explicit estimates of potential forest biomass worldwide. The first set of analyses was based on ‘bottom-up’ models built directly from ground-sourced (denoted GS) aboveground live biomass estimates from forest inventory data of the Global Forest Biodiversity initiative (GFBI)^6^. This was contrasted with three ‘top-down’ models built from the latest satellite-derived (denoted SD), high-resolution aboveground forest biomass maps, namely, the European Space Agency’ Climate Change Initiative (ESA-CCI)^7^, Walker et al.^2^ and harmonized^8^ products. As our GS model operates independently from satellite information, it serves as a benchmark for evaluating the satellite-driven approaches. For all four datasets, we then approximated forest biomass under hypothetical natural conditions through two distinct methods: (1) representing human-disturbance indices as independent variables (model type 1) and (2) building models exclusively using data from undisturbed areas (model type 2). We define ‘natural’ forest potential as that which might exist in the absence of extensive anthropogenic degradation. Using each of these databases, we then scaled to total forest carbon potential using spatially explicit global estimates of root mass fraction^24^, soil carbon potential^23^ and biome-level estimates of dead wood and litter^19^. By contrasting these diverse approaches and comparing the results against previous evaluations using a meta-analysis, we aimed to provide an integrated assessment of the natural forest carbon potential.

## Mapping the human impact on tree biomass

**Fig. 1:**
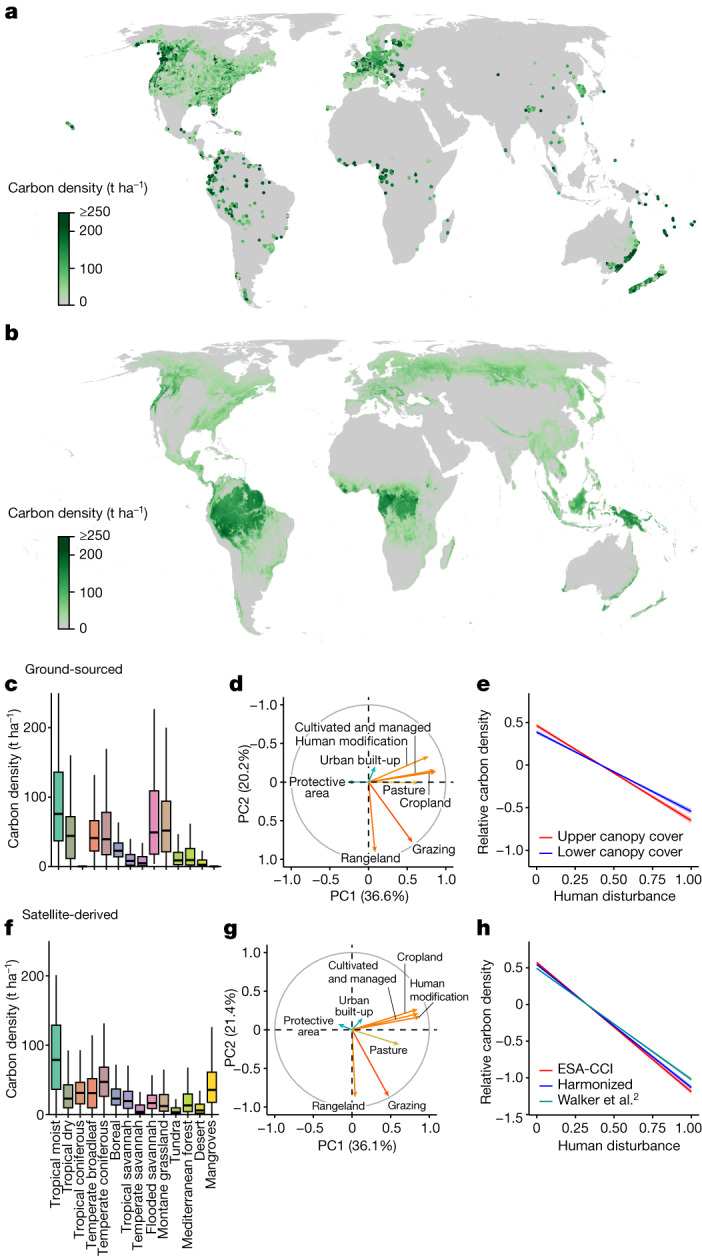
The global distribution of tree carbon observations and the impact of human disturbances. **a**, Map of ground-sourced aboveground tree carbon observations (GFBI data; aggregated to 30-arcsec (1-km^2^) resolution). **b**, Satellite-derived ESA-CCI map of current aboveground tree carbon stocks (1-km resolution). **c**,**f**, Observed biome-level tree carbon densities in existing forests based on ground-sourced (**c**) and satellite-derived (**f**) data. **d**,**g**, Principal component analysis (top two principal components shown) of the eight human-activity variables either directly or indirectly reflecting human-caused forest disturbances or the lack thereof, such as land-use change, human modification, cultivated and managed vegetation and wilderness area, to detect the effect of human disturbance on tree carbon densities for the ground-sourced (**d**) and satellite-derived data (**g**). **e**,**h**, Partial regression of the global variation in forest carbon density along the human-disturbance gradient (represented by the first principal component of the eight human-activity variables; see panels **d** and **g**) for the ground-sourced (**e**) and satellite-derived data (**h**), controlling for 40 environmental covariates. Relative carbon density is the observed carbon density divided by the global average.

The underlying goal of our analysis was to investigate the impact of human land-use change on forest carbon stocks globally. Of course, many indigenous populations and local communities live in sustainable harmony with natural forests, often with beneficial impacts on ecosystem structure. However, we aimed to isolate the effects of extensive land-use change and anthropogenic degradation. To achieve this, we used a partial-regression approach in the first step, testing for the relationship between aboveground forest biomass and anthropogenic degradation, while controlling for the effects of climate, topography and soil conditions (Fig. 1d,g and Methods). This analysis revealed a consistent decline in tree carbon density along the anthropogenic degradation gradient across all biomes, evident in both the ground-sourced and the satellite-derived biomass observations (Fig. 1e,h).

Our GS models of potential forest biomass combine plot-level aboveground forest carbon measurements with spatially explicit data reflecting climate, soil conditions, topography, forest canopy cover and human disturbance, using random-forest machine-learning models to interpolate our biomass measurements across the globe (see Methods). In the first set of models (GS1), we estimated the global forest carbon potential in the absence of human activity by statistically accounting for the impact of human disturbance^23^, setting all variables directly reflecting human disturbance to zero. By contrast, the second set of GS models (GS2) extrapolated the global forest carbon potential from data derived from protected areas with minimal human disturbance^2,3^. To account for uncertainties in canopy-cover estimates from the forest inventory plots, we incorporated upper and lower boundaries of canopy cover in each pixel, resulting in a total of four GS models: GS1_Upper_, GS1_Lower_, GS2_Upper_ and GS2_Lower_. We extended this combination of approaches to evaluate the biomass potential for each of the three satellite-derived biomass products (ESA-CCI, Walker et al. and harmonized). The models included either all terrestrial regions (SD1) or only regions with minimal human disturbance (SD2), using the same set of predictor variables as covariates included in the GS models. This resulted in a total of six SD models: SD1_ESA-CCI_, SD1_Walker_, SD1_Harmonized_, SD2_ESA-CCI_, SD2_Walker_ and SD2_Harmonized_.

**Fig. 2:**
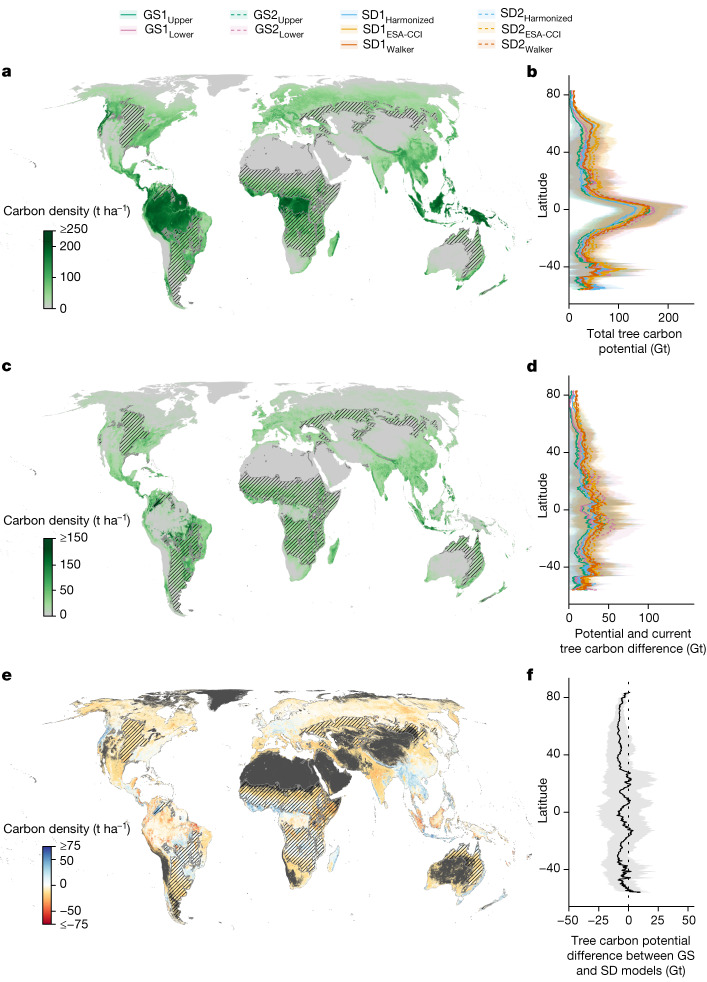
The natural tree carbon potential under current climate conditions in the absence of humans. **a**,**b**, The total living tree carbon potential of 600 Gt C within the natural canopy cover area of 4.4 billion ha^2^. **c**,**d**, The differences between current and potential tree carbon stocks, totalling 217 Gt C. **e**,**f**, The difference of tree carbon potential between the GS and SD models, subtracting the mean values of the six SD models from the mean values of the four GS models. Blue colours indicate that the GS models predict higher potential than the SD models, whereas red colours indicate the opposite. **b**,**d**,**f**, Latitudinal distributions (mean ± standard deviation) of the total tree carbon potential for the GS1, GS2, SD1 and SD2 models (**b**), the difference between current and potential tree carbon (**d**) and the difference of tree carbon potential between the GS and SD models (**f**). Maps represent the average estimates across all GS and SD models and are projected at 30-arcsec (about 1-km^2^) resolution. We show dryland and savannah biomes with stripes to denote that many of these areas are not appropriate for forest restoration. Where trees would naturally exist, they often exist far below 100% canopy cover, and restoration of forest cover should be limited to natural conditions.

The full combination of models allowed us to disentangle the effects of deforestation and forest degradation on tree carbon losses while representing data and model uncertainties. The total tree carbon potential was determined by summing the forest carbon that would naturally exist (1) outside existing forests (restoration potential) and (2) in existing, degraded forests (conservation potential). The resulting maps provide models of tree carbon potential under current (1979–2013) climate conditions in the hypothetical absence of human disturbance (Fig. 2a).

The coefficients of variation from a bootstrapping procedure showed that existing and potential carbon stocks were estimated with confidence across all models. For 90–100% of the pixels inside the existing and potential forest area, the coefficients of variation were below 20% (Supplementary Figs. 1 and 2). A spatial-validation procedure (spatially buffered leave-one-out cross-validation (LOO-CV)), accounting for the potential effects of spatial autocorrelation on model-validation statistics, showed that the GS and SD models explained 70–77% or 82–87% of the spatial variation in tree biomass, respectively (Supplementary Table 1 and Supplementary Fig. 3). Furthermore, when specifically considering disturbed regions with human-disturbance levels ranging from 10% to 60%, the explained variation in tree biomass remained high (>60%), showing that our models effectively captured the variation of carbon stocks in regions with high human footprint (Supplementary Fig. 4).

## Comparison between models

Despite discrepancies in certain regions, there was high overall agreement between the ground-sourced and satellite-derived biomass estimations at the global scale (average *R*^2^ of 0.72 at a spatial resolution of approximately 1 km^2^; Supplementary Figs. 5–9). This agreement translated to similar estimates of existing live tree biomass: 367 Gt C (model range = 334–400 Gt C) for the GS models and 394 Gt C (model range 355–445 Gt C) for the SD models (<7% difference). A comparison of existing biomass estimates across the latitudinal gradient also showed high inter-model consistency, with the GS model predicting slightly higher biomass values than the SD model for the equatorial zone and lower biomass values at high-latitude regions of the Southern Hemisphere (>40 °S) (Supplementary Fig. 6). On average, the models predicted that 69% of live tree biomass is stored in tropical regions, with temperate, boreal and dryland regions accounting for 18%, 11% and 1%, respectively (Supplementary Table 3).

**Fig. 3:**
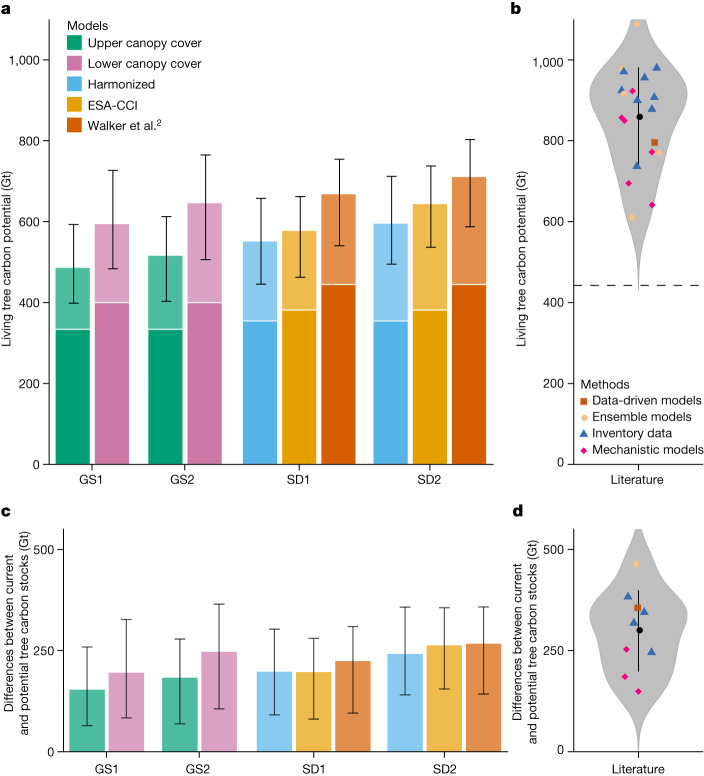
The living tree carbon potential estimated from the ground-sourced (GS1 and GS2) and satellite-derived (SD1 and SD2) models. **a**, Total estimated living tree biomass potential of the GS1, GS2, SD1 and SD2 models. Error bars represent the lower and upper boundaries based on the 5% and 95% quantiles from a bootstrapping procedure. Colours represent the different input datasets, that is, upper or lower canopy cover boundaries (GS models) and ESA-CCI, Walker et al.^2^ or harmonized (SD models). Light colours above white lines indicate the difference between current and potential tree carbon stocks. **b**, Meta-analysis showing literature estimates of living tree carbon potential based on ensemble models^4,53,54^, inventory data^19,55–61^ and mechanistic^62–67^ or data-driven^2^ models. The horizontal dashed line represents the average existing living tree carbon of 443 Gt C estimated in these publications. **c**, Differences between current and potential tree carbon stocks. **d**, Literature estimates for the difference between current and potential tree carbon stocks from ref. ^4^ (ensemble models), refs. ^1,53,58,61^ (inventory data), refs. ^63,64^ (mechanistic models) and ref. ^2^ (data-driven models).

**Fig. 4:**
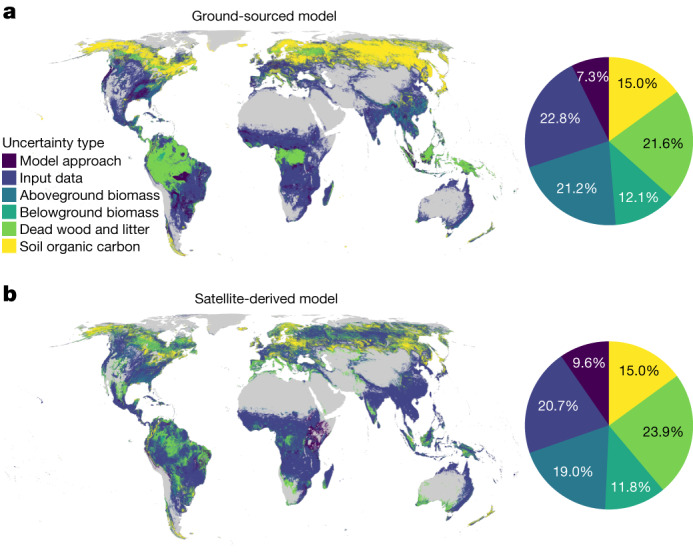
Sources of uncertainty in forest carbon potential for the GS and SD models. **a**,**b**, Relative contribution of individual uncertainty sources to the overall uncertainty in carbon potential for the GS (**a**) and SD (**b**) models: (1) model approach (type 1 versus type 2 models); (2) input data (current aboveground tree carbon input, that is, upper and lower canopy cover boundaries for GS models and ESA-CCI, Walker et al.^2^ and harmonized for SD models); (3) aboveground biomass potential estimates (bootstrapping); (4) belowground biomass (accounting for uncertainties in both root mass fraction and aboveground biomass); (5) dead wood and litter (accounting for uncertainties in both dead wood and litter-to-tree biomass ratios and tree biomass); and (6) soil organic carbon potential^23^. The maps show the top uncertainty source within each pixel. The pie charts show the relative contribution of uncertainties worldwide.

**Fig. 5:**
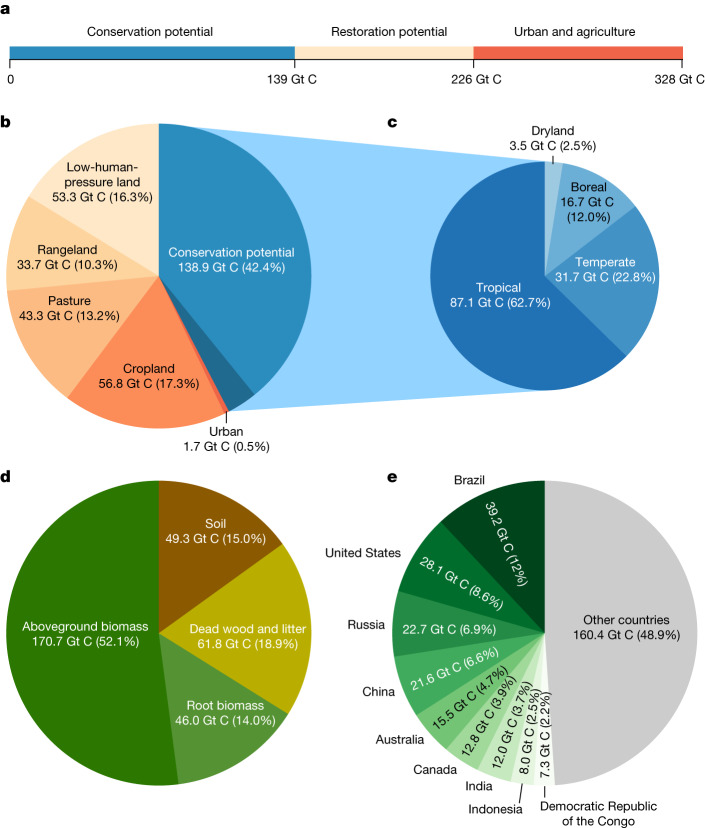
Contribution of land-use types, forest types, carbon pools and countries to the difference between current and potential ecosystem-level carbon stocks. **a**, Of the 328 Gt C discrepancy between current and potential carbon stocks, 226 Gt C is found outside urban and agricultural (cropland and pasture) areas, with 61% in forested regions in which the recovery of degraded ecosystems can promote carbon capture (conservation potential) and 39% in regions in which forests have been removed (restoration potential). **b**, Relative contribution of forest degradation (conservation potential; blue area) and land-cover change (orange colours) to the difference between current and potential ecosystem-level carbon stocks. The darker blue area represents the conservation potential of 10.5 Gt C in forest plantation regions. **c**, Relative contribution of tropical, temperate, boreal and dryland forests to the total forest conservation potential. **d**, Relative contribution of the three main carbon pools (living biomass, dead wood and litter, and soil) to the difference between current and potential carbon stocks. **e**, The nine countries contributing more than 50% to the difference between current and potential carbon stocks.

Using all sets of GS and SD models, we could estimate the total potential living tree carbon that would exist in the absence of human influence. Our models projected considerable gains in the hypothetical natural forest biomass, with a mean estimate for total potential living tree carbon of 600 Gt C (model range = 487–712 Gt C). The individual model estimates were as follows: GS1_Upper_ = 487 Gt C, GS1_Lower_ = 595 Gt C, GS2_Upper_ = 517 Gt C, GS2_Lower_ = 647 Gt C, SD1_Harmonized_ = 552 Gt C, SD1_ESA-CCI_ = 578 Gt C, SD1_Walker_ = 669 Gt C, SD2_Harmonized_ = 596 Gt C, SD2_ESA-CCI_ = 645 Gt C and SD2_Walker_ = 712 Gt C (Figs. 3 and 4 and Supplementary Tables 2 and 3). The highest estimates were derived from the Walker et al.^2^ map, with the GS, harmonized biomass and ESA-CCI estimates being 19%, 17% and 11% lower, respectively. Overall, we predict that, under current climate conditions, a further 217 Gt (model range = 153–267 Gt) of living tree carbon could potentially exist in the absence of humans (Fig. 5b). Of this potential, 123 Gt C (99–153 Gt C) can be attributed to tropical regions, 55 Gt C (40–66 Gt C) to temperate regions, 14 Gt C (5–25 Gt C) to boreal regions and 25 Gt C (9–41 Gt C) to dryland regions (Supplementary Table 3).

Despite the broad consensus on the global top-down and bottom-up carbon potential estimates, considerable spatial variations were observed in the models. The SD models tended to predict higher potential carbon stocks than the GS models across 82% of pixels, particularly in South American tropical forests (Fig. 2e,f), suggesting possible overestimation of satellite-derived biomass potential in these regions. More ground-sourced data are needed from tropical areas to improve accuracy and balance the high sample sizes available for temperate regions^7,25^. On the other hand, the GS models predicted slightly higher potential than the SD models in subtropical regions and temperate forests of Europe.

We also show that the type 1 models (GS1 and SD1) predicted a 47 Gt C lower potential than the type 2 models (GS2 and SD2; Fig. 3). The focus on ‘undisturbed’ regions in the type 2 models may introduce bias by favouring regions with unusually high biomass. By contrast, the type 1 models incorporated observations across the full human-disturbance gradient, potentially resulting in an underestimation of potential in regions with incomplete historic-disturbance data. Furthermore, we imposed a constraint on forest biomass potential by limiting forest growth to the potential tree cover range projected in a previous analysis^3^. If this spatial constraint is removed to compare our model with the estimate of Walker et al.^2^ of 796 Gt C (without such constraints), our SD2_Walker_ model generates a similar total potential of 760 Gt C (<5% difference). Thus, our mean estimate of Earth’s total potential living tree carbon of 600 Gt C from the ensemble of modelling approaches is probably conservative.

## Total ecosystem carbon potential

To determine the total carbon storage potential of natural woody ecosystems, we converted our estimates of living tree biomass into total ecosystem carbon stocks by incorporating global data on soil carbon^23^, dead wood and litter^19^. To represent the various sources of uncertainty (Fig. 4), we considered: (1) model type (types 1 and 2); (2) input data (upper and lower canopy cover boundaries for GS models; ESA-CCI, Walker et al. and harmonized for SD models); (3) aboveground biomass potential (bootstrapping); (4) tree root biomass; (5) dead wood and litter; and (6) soil carbon^23^. The GS and SD models exhibited similar uncertainty contributions globally, with 21.2% and 19.0% attributed to aboveground living tree biomass potential, 21.6% and 23.9% to dead wood and litter, 22.8% and 20.7% to aboveground biomass input data, 15.0% to soil carbon, 12.1% and 11.8% to root biomass and 7.3% and 9.6% to model type. Soil carbon emerged as the primary source of uncertainty in regions with high latitudes and elevation. By contrast, aboveground biomass input data and dead wood and litter were the primary sources of uncertainty in dry and humid tropical areas, respectively (Fig. 4).

Considering all carbon pools together, we estimate that current forest carbon storage is 328 Gt (221–472 Gt) lower than the full natural potential (Fig. 5 and Table 1). Of this difference, 226 Gt C (151–363 Gt C) exist outside urban and agricultural areas, with 61% in forested regions in which sustainable management and conservation can promote carbon capture through the recovery of degraded ecosystems and 39% in regions in which forests have been removed (Table 1). These estimates highlight that forest conservation, restoration and sustainable management can help achieve climate targets by mitigating emissions and enhancing carbon sequestration.

**Table 1:** Differences between current and potential carbon stocks for living tree biomass, dead wood and litter, soil (0–2 m depth) and total ecosystem carbon in different land-use types

Land-use types	Living tree biomass	Dead wood and litter	Soil	Total
Urban	1.1 (0.8–1.4)	0.3 (0.2–0.4)	0.3 (0.2–0.5)	1.7 (1.2–2.2)
Cropland	38.3 (25.5–50.7)	10.1 (6.7–13.3)	8.5 (5.4–12.6)	56.8 (37.5–76.6)
Pasture	29.8 (21.0–36.5)	7.6 (5.3–9.2)	6.0 (3.5–9.4)	43.3 (29.8–55.1)
Rangeland	24.6 (11.4–38.7)	6.1 (2.8–9.5)	3.1 (1.9–4.6)	33.7 (16.0–52.9)
Low-human-pressure land	37.0 (18.8–54.4)	12.2 (6.0–17.2)	4.2 (2.4–10.5)	53.3 (27.2–82.1)
Existing forest	84.7 (68.7–105.0)	26.9 (25.9–32.3)	27.3 (13.5–91.1)	138.9 (108.1–228.4)
Sum	216.7 (153.0–266.8)	61.8 (41.3–76.2)	49.3 (26.8–128.7)	327.8 (221.1–471.7)

Values show the means (in Gt C) of the four GS and six SD model predictions. Values in brackets show the full range of estimates across the ten models. For soil carbon, the uncertainty range (absolute errors) was based on ref. ^23^.

## Carbon potential in existing forests

Previous work has suggested that up to 80% of the world’s forests are secondary systems that have undergone anthropogenic degradation^26^. Our models corroborate these findings, revealing a considerable potential for carbon capture in existing forests by allowing these degraded ecosystems to regenerate to maturity. The difference between current and potential ecosystem carbon stocks amounts to 139 Gt C (108–228 Gt C) in existing forests, representing 61% of the total difference when excluding urban and agricultural areas (Table 1). Of the total 139 Gt, 11 Gt (8%) can be attributed to biomass loss in existing forest plantations, in which restoring diverse ecosystems could lead to further carbon capture. The remaining 128 Gt can be attributed to human degradation in other forest ecosystems. These findings highlight the importance of forest conservation for carbon capture, as ecosystems are allowed to recover to their mature states. It suggests that a substantial proportion of carbon capture can be achieved with minimal land-use conflicts. However, it is essential to acknowledge that the demand for wood and other forest-based products imposes limitations on this potential, given their climate benefits as substitutes for carbon-intensive materials such as fossil fuels and concrete^5^. Nonetheless, evidence shows that reductions in harvesting intensity and forest degradation can deliver important climate benefits^27^. Moreover, our model might underestimate the extent of degradation owing to challenges in capturing historical land-use legacies and limited data availability on plantations in certain countries^28^. These observations reinforce the importance of effective forest conservation and management not only in reducing future carbon emissions^15,29^ but also in removing carbon that has already been released into the atmosphere.

## Carbon potential in converted lands

In areas in which forests have been removed, the difference between the current and potential forest carbon stocks amounts to 189 Gt C (112–269 Gt C). Of this difference, 30% (57 Gt C) can be attributed to cropland areas, 28% (53 Gt C) to areas experiencing low anthropogenic pressure at present, 23% (43 Gt C) to pasture land, 18% (34 Gt C) to rangeland and 1% (2 Gt C) to urban areas (Fig. 5, Table 1 and Supplementary Fig. 10). It is important to recognize that the scale of this potential is contingent on social land-use constraints. Socially responsible ecosystem restoration must be driven by the land-use decisions of local communities, especially indigenous communities that often face marginalization. Sustainable economic development that promotes approaches that work with nature (for example, agroforestry, ecotourism etc.) can provide critical avenues for long-term financial security as a result of healthy nature. Also, it is important to acknowledge that forests can lead to reductions in surface albedo^30,31^, which generally have warming effects in high-latitude regions. Conversely, the local biophysical cooling effects of forests in warmer regions^32^ probably enhance the climate-adaptation benefits in the global south.

Taking into account the future food and feed demand, the Intergovernmental Panel on Climate Change (IPCC) highlights a range of measures to improve ecosystem health and carbon storage in the land-use sector^33^. This will require a diverse range of approaches, including sustainable diets, reducing food waste, rewetting, improved soil health, methane reduction and promoting the use of wood in construction. We estimate that approximately 41% of the difference between current and potential ecosystem carbon stocks outside existing forests, within the areas of the world that would naturally be forested, can be attributed to livestock grazing areas (pasture and rangelands). Also, 36% of the world’s crop yields are being used for animal feed^34^. This impact of animal husbandry on forest ecosystems underscores the potential implications of transitioning to more plant-based diets. Besides reducing greenhouse gases that directly stem from animal farming (methane emissions, food production), a reduction in meat consumption could reduce emissions from land-use change and create large carbon sinks if ecosystems were allowed to regenerate on former pasture lands^35,36^.

## Comparison with previous estimates

Our integrated estimate of the difference between current and potential global living tree biomass (217 Gt C) falls at the lower end of the range of previous estimates, which ranged from 150 to 446 Gt C (Fig. 3c,d). Also, our estimate of the extra potential for total ecosystem carbon storage outside urban and agricultural land (226 Gt C) aligns closely with recent global-scale estimates of 205 and 287 Gt C (refs. ^2,3^). However, it is worth noting that three previous data-driven approaches, not included in this meta-analysis because of methodological differences, have suggested carbon potential values below this range. Specifically, Lewis et al.^9^ considered more rigorous social constraints and estimated that natural restoration of 350 Mha of deforested, tropical land could capture 42 Gt C in living tree biomass. Scaling this estimate to 900 million hectares yielded a potential of 89–108 Gt tree carbon^20^, which is comparable with our estimate of tree biomass restoration potential of 91 Gt C outside existing forest, urban areas and cropland regions (Table 1). Similarly, Roebroek et al.^5^ recently reported that the carbon potential in existing forests could be as low as 44 Gt C. Their estimate is considerably lower than our conservation potential estimate of 139 Gt C. This difference arises because Roebroek et al.^5^ focused only on aboveground tree biomass (excluding soil, roots, dead wood and leaf litter) and only considered the tree cover of existing forested regions. When we narrow our analysis to aboveground biomass in these forests, we recover a similar estimate of forest potential of 50 (39–63) Gt C. Nonetheless, when we consider studies that focused on the total ecosystem potential in all forest regions, our analysis reveals a distinct overlap that provides confidence in the scale of carbon losses from the global forest system.

## Discussion

Understanding the potential for carbon storage in natural forests is crucial for comprehending their role in combating climate change. Our combined modelling approach, including ten estimates from this study and nine others from previous studies, allows us to identify the extent of overlap across diverse approaches and increases our confidence about the scale of the forest carbon potential across the globe. We found that total forest carbon storage is, at present, 328 Gt C (model range = 221–472 Gt C) below its full potential. Of this potential, 102 Gt C (69–134 Gt C) exist in urban areas, cropland and permanent pasture sites, in which substantial restoration is highly unlikely. Yet, a potential of 226 Gt C (151–363 Gt C) is in existing forests and regions with low human pressure (Table 1). Of this constrained forest carbon potential, 139 Gt C (61%) can be found in regions that are already forested. This highlights that the prevention of deforestation does not only contribute to the reduction of carbon emissions but has large carbon drawdown potential if ecosystems can be allowed to return to maturity. Improved forest management and restoration to reconnect fragmented forest landscapes contribute a considerable 87 Gt (39%) to the extra carbon drawdown potential. We stress that, despite considering the broad land-use types, we cannot identify detailed land-use activities at a high resolution, so different social and economic considerations may place further constraints on the scale of this potential. Nevertheless, this work highlights the potential contribution of forest conservation, restoration and sustainable management in capturing carbon from the atmosphere.

The development of current and natural forest carbon maps involved several approaches and data sources with varying strengths and weaknesses. This ensemble of modelling approaches can help to identify the extent of agreement and uncertainty across modelling approaches, enabling a comprehensive understanding of carbon potential at a global scale. As new satellite technologies, such as the Global Ecosystem Dynamics Investigation (GEDI) project^37^, begin to reveal high-resolution information about forest structure, it will be increasingly important to refine the spatial and temporal resolution of these carbon stock models. Our multimodel and multidata comparison pinpoints regional variation in the main sources of uncertainty in forest carbon potential, highlighting the need for improved aboveground data-sampling efforts in the tropics and soil carbon sampling at high latitudes (Fig. 4). As such, continuing efforts to refine the confidence in this forest carbon potential require advancements in remote-sensing instrumentation^7^, field-monitoring strategies with sustained funding for research teams and field workers, especially in the Global South^38,39^, better representation of temporal dynamics in carbon stocks, especially in ecosystems prone to natural disturbances^40^, and methodology to allow for strict and verifiable integration of ground data and remote sensing into comprehensive carbon stock estimates^41^. Fair and equitable funding support for sustaining and sharing tropical forest data is vital to reduce global sampling biases in forest inventory efforts^38,39^ (Supplementary Fig. 11).

It is important to note that our estimates of potential carbon capture in woody ecosystems pertain only to the biophysical potential and do not account for future changes in human pressure that may threaten forests^42,43^. Moreover, our estimations are based on recent climate conditions (1979–2013). If fossil fuel emissions continue to rise, the capacity of ecosystems to capture and store carbon will be threatened by climate-change-induced factors such as increasing temperature, drought and fire risks^44,45^. CO_2_ fertilization also has the potential to further change this system^46^. The dynamic and vulnerable nature of forests underscores the urgency of conserving existing ecosystems to maintain their carbon sink potential and highlights the urgent need to uphold no-deforestation pledges at the 26th UN Climate Change Conference of the Parties (COP26), including public and private-sector commitments to end forest loss as soon as 2025 (refs. ^18,47,48^).

Given the positive effect of biological diversity on ecosystem productivity^6,49^, the magnitude of the estimates presented here can only be achieved in ecosystems that support a natural diversity of species. Indeed, almost half of global forest production can be directly or indirectly attributed to the role of biodiversity^6^, highlighting that the full carbon potential cannot be achieved without a healthy diversity of species. Ecologically responsible forest restoration does not include the conversion of other natural ecosystem types, such as grasslands, peatlands and wetlands, that are equally essential. Restoration can take many forms, including the protection of land to allow natural vegetation recovery, soil microbiome enhancement, enrichment planting or reintroducing wild animals^33,50^. It also includes a vast array of active management practices, such as sustainable agroforestry, silviculture or permaculture practices, to promote biodiversity in managed systems. Ultimately, the protection and restoration of forest ecosystems are complex social, political and economic challenges that require the development of land-management policies that give priority to the rights and wellbeing of local communities and indigenous people^51^. Only when healthy biodiversity is the preferred choice for local people can ecosystem-restoration initiatives be sustainable in the long term^52^. When built in a socially and ecologically responsible way, the promotion of diverse forests can contribute substantially to achieving our combined climate and biodiversity goals.

## Methods

### Ground-sourced tree biomass

#### Forest inventory data

Plot-level forest inventory records were obtained from data compiled in the GFBI database^6^ (http://www.gfbinitiative.org), which hosts information for 1,188,771 plots (median plot size = 250 m^2^) from every continent except Antarctica (Fig. 1). Each plot contains information on stem diameter at breast height (DBH) for each tree^6^. Individuals with a DBH < 5 cm were removed from the analysis. Quality controls of tree density values were conducted and we removed plots with tree densities that fell outside the median ± 2.5 times the median absolute deviation (moderately conservative threshold)^68^ in each biome (6% of total plots). This resulted in retaining a total of 25,779,993 tree observations in 1,089,026 plots.

#### Biomass estimation for individual trees

For extratropical biomes, we used 430 species-specific DBH-based allometric equations obtained from the GlobAllomeTree database^69^ to estimate the aboveground biomass of each tree, *W*. These allometric equations use a common logarithmic equation for estimating aboveground biomass from DBH measures^70^:

1 $${\rm{ln}}(W)={\beta }_{0}+{\beta }_{1}\times {{\rm{ln}}}^{{\rm{DBH}}}$$

in which *W* is the predicted individual aboveground biomass (kg dry weight), DBH is the measured diameter at breast height (cm), ln is the natural logarithm and *β*_0_ and *β*_1_ are the parameter estimates.

Following ref. ^70^, we applied back calculation to generate a pseudo dataset for biomass changes along DBH gradients based on each of the 430 allometric equations. To generate the pseudo data, we applied the following rules: (1) for a DBH between 5 and 25 cm, each centimetre was assigned a corresponding pseudo biomass value; (2) for a DBH between 25 and 100 cm, every 5 cm was assigned a corresponding value; (3) for a DBH between 100 and 300 cm (maximum DBH), every 10 cm was assigned a corresponding value. We then trained biome-specific allometric equations (varying in the *β*_0_ and *β*_1_ parameter estimates) based on the pseudo DBH and biomass dataset^71^ (Supplementary Fig. 12 and Supplementary Table 4).

Biomass estimations for the tropics followed the allometric model for pantropical regions from ref. ^72^, which is available through the R package BIOMASS (ref. ^73^). These equations require information on wood density, which came from the Global Wood Density Database^74^ and the Biomass And Allometry Database (BAAD)^75^. To match the binomial species names between the GFBI and the wood density databases, we standardized species binomials using the Taxonomic Name Resolution Service (TNRS)^76^.

#### Plot-level tree biomass calculation

After computing the aboveground dry biomass for all approximately 28 million individuals in our dataset, plot-level biomass values were obtained by summing the biomass of all individuals in the respective plot. For plots that contained data for several years, we calculated the mean of these years. The median year of observation across all plots was 2002. Subsequently, the biomass densities (in t ha^−1^) of each plot were obtained by dividing the total aboveground biomass (*W*) by the plot area. Carbon values were obtained by multiplying tree biomass by biome-specific wood carbon concentrations, ranging from 45.6% in tropical moist broadleaf forest to 50.1% in temperate conifer forest^77^ (see Supplementary Table 5). The spatial modelling was performed at 30-arcsec (about 1-km^2^) resolution and we therefore averaged tree carbon-density values for plots located in the same 30-arcsec pixel.

To avoid overestimation of carbon densities, we removed (1) values larger than the maximum carbon density ever recorded for forests (1,867 t C ha^−1^) and (2) values that fell outside the median ± 2.5 times the median absolute deviation (moderately conservative threshold) in each biome^68,78^. Small outlier values were kept, however, if they fell in human-modified non-forest landscapes, that is, regions with a human-disturbance index >10% and canopy cover <10%. This was done to avoid the underestimation of current carbon in croplands, pasture lands and urban areas that can contain notable amounts of existing biomass in trees outside forests^79^. To obtain normally distributed data, the carbon-density values were log-transformed before the median absolute deviation was calculated, using the following equation (Supplementary Fig. 13):

2 $${x}_{{\rm{transformed}}}=\log (x+1)$$

This removed 6.4% of the data (0–6% in biomes), resulting in a total of 527,767 spatially distinct carbon-density values used for the final analysis.

### Environmental and human-disturbance variables

#### Environmental covariates

In total, 40 layers, reflecting climate, soil and topographic features, were used as covariates in our analyses (Supplementary Table 6). All layers were standardized to 30-arcsec resolution (1 km^2^ at the equator). Layers for 19 bioclimatic variables came from the CHELSA version 1.2 open climate database (www.chelsa-climate.org)^80^, topographic information (elevation, slope, roughness, eastness, northness, aspect cosine, aspect sine and profile curvature) from the EarthEnv (www.earthenv.org/topography) database^81^, cloud cover (annual mean, inter-annual standard deviation and intra-annual standard deviation) from the EarthEnv (www.earthenv.org/cloud) database and ref. ^82^, depth to the water table from ref. ^83^, the annual mean of solar radiation and wind speed from the WorldClim database (version 2)^84^, absolute depth to bedrock and soil texture (clay content, coarse fragments, sand content, silt content and soil pH), averaged for the depth between 0 to 100 cm below surface, from the SoilGrids database^85^ and the Global Aridity Index from the Global Aridity Index and Potential Evapotranspiration (ET0) Climate Database version 2.0 (refs. ^86,87^).

#### Human-disturbance covariates

To represent human disturbance in our models, we used eight global layers that directly reflect anthropogenic effects on the environment. Information on the proportion of cultivated and managed vegetation and urban built-up areas in each pixel came from the EarthEnv database^88^. These maps integrate four global land-cover products to represent accuracy-weighted consensus information on the prevalence of land-cover classes at 1-km resolution across the globe (except for Antarctica). By representing the proportional area of anthropogenic modification in each pixel (urban area or managed vegetation), the maps provide information on the spatial extent of human disturbance within pixels.

Information on agricultural land use (cropland, grazing, pasture and rangeland layers transformed to the percentage of agricultural land in each pixel) came from the HYDE database version 3.1 (refs. ^89,90^). Each layer represents the proportional area of cropland, grazing, pasture or rangeland in each pixel, thus allowing us to account for the individual impacts of agricultural land-use types.

Information on human modification, reflecting the overall intensity of human activity, came from ref. ^91^. Rather than representing the impact of individual human-modification classes, such as urban areas or cropland, this map provides a cumulative measure of human modification based on models of the physical extent of 13 anthropogenic stressors in five main classes: (1) human settlement (population density, built‐up areas); (2) agriculture (cropland, livestock); (3) transport (major roads, minor roads, two tracks, railroads); (4) mining and energy production (mining, oil wells, wind turbines); and (5) electrical infrastructure (power lines, night-time lights).

All variables were scaled to represent a continuous gradient of human impact, whereby values of zero indicate no human impact in the respective pixel and values of 1 indicate maximum human impact. Also, we included information on the regions with minimal human disturbance across the globe, using the global protected area map from the World Database on Protected Areas^92,93^. Protected areas were treated as a binary variable of whether the respective pixel is intentionally disturbed by humans or not (that is, strict nature reserve or wilderness area)^94^.

### Geospatial modelling of existing tree carbon

#### Ground-sourced tree carbon density model

To represent the uncertainty in canopy cover of the forest inventory plots, we used upper and lower boundaries of canopy cover in each pixel at approximately 30-m resolution to convert C per plot to C per pixel^95^. We either assumed the canopy cover (% forested) of each forest inventory plot to represent the maximum canopy cover observed for the respective 1-km^2^ pixel (termed ‘upper canopy cover estimate’) or the mean canopy cover of the forested part (≥10% canopy cover) of the respective pixel (‘lower canopy cover estimate’). This canopy cover range ensured that our estimates represent the range of feasible sampling designs, as forest inventory plots can be biased towards high canopy cover sites within pixels rather than representing the average forest canopy cover. To convert C per plot into C per pixel, we divided the C per plot by the canopy cover within the plot (assuming either upper or lower canopy cover) and multiplied by canopy cover of the entire pixel, that is, C per pixel = (C per plot)/(canopy cover within plot) × (% forested per pixel). Thus, the resulting carbon value is inversely related to the canopy cover of the forest inventory plots: if the plot locations are assumed to reflect the maximum canopy cover in the pixel, then the resulting carbon estimate is the smallest; if instead the plots reflect the mean canopy cover, then the resulting carbon estimate is the largest. Note that we do not consider the scenario in which the plots are preferentially located in areas with minimum forest canopy cover, as this would lead to unrealistically high pixel-level carbon estimates (and carbon potential values) and is also unrealistic given the study design of the forest inventories underpinning the data. All subsequent analyses were conducted using C per pixel derived from both the upper and lower plot-level canopy cover estimates, allowing us to represent the uncertainty associated with canopy cover.

To train spatially explicit tree carbon models across the world’s forests, we ran random-forest machine-learning models using Google Earth Engine^96^. The models included 40 environmental layers (representing climate, soil and topographic features), eight human disturbance layers, and canopy cover as predictors. In random forest, unlike traditional regression, correlation among variables does not affect the model accuracy. Indeed, the ability to use many correlated predictors is one of the key benefits of machine-learning models^97^. When variables are correlated, the effect of these variables is ‘shared’ across the trees in the random forest. Because random forest does not estimate coefficients as in regression, this correlation does not hinder model fit or performance but, rather, complicates efforts to quantify variable importance, which is also shared across correlated variables (see Supplementary Fig. 14 for an evaluation of variable importance using a reduced, uncorrelated set of variables). Thus, including numerous variables, even if correlated, can improve the predictive power of the model to accurately quantify current carbon.

The model had the following form:

3 $${C}_{{\rm{C}}{\rm{u}}{\rm{r}}{\rm{r}}{\rm{e}}{\rm{n}}{\rm{t}}}=\mathop{\sum }\limits_{i=1}^{n}{\overrightarrow{\alpha }}_{i}{\mathop{{\rm{V}}{\rm{a}}{\rm{r}}}\limits^{\longrightarrow }}_{i}^{{\rm{E}}{\rm{n}}{\rm{v}}}+\mathop{\sum }\limits_{j=1}^{m}{\overrightarrow{\beta }}_{j}{\mathop{{\rm{V}}{\rm{a}}{\rm{r}}}\limits^{\longrightarrow }}_{j}^{{\rm{H}}{\rm{u}}{\rm{m}}{\rm{a}}{\rm{n}}}+\overrightarrow{\gamma }{\mathop{{\rm{V}}{\rm{a}}{\rm{r}}}\limits^{\longrightarrow }}^{{\rm{C}}{\rm{a}}{\rm{n}}{\rm{o}}{\rm{p}}{\rm{y}}{\rm{C}}{\rm{o}}{\rm{v}}{\rm{e}}{\rm{r}}}$$

in which *C*_Current_ is the current forest tree carbon density in each pixel, ${\mathop{{\rm{V}}{\rm{a}}{\rm{r}}}\limits^{\longrightarrow }}^{{\rm{Env}}}$ are the environmental variables, ${\mathop{{\rm{V}}{\rm{a}}{\rm{r}}}\limits^{\longrightarrow }}^{{\rm{Human}}}$ are the variables directly representing human disturbance (see ‘Environmental and human-disturbance variables’ section for details) and ${\mathop{{\rm{V}}{\rm{a}}{\rm{r}}}\limits^{\longrightarrow }}^{{\rm{CanopyCover}}}$ is the current canopy cover for the year 2010 (ref. ^95^).

In a first step, we tested for the existence of spatial autocorrelation in model residuals, which can bias model-validation statistics^98^. This was done by calculating the Moran’s *I* index of the residuals from generalized additive models at different spatial scales (0–1,000 km). The Moran’s *I* indices indicated residual spatial autocorrelation at distances of up to 80 km for all GS models (Supplementary Fig. 15a–d). To avoid any bias introduced by the influence of spatial autocorrelation and correct for the uneven sampling across regions, we therefore applied bootstrapped spatial subsampling (100 iterations) to predict both current and potential tree carbon densities (see ‘Geospatial modelling of tree carbon potential’ section). The spatial subsampling was conducted by subsampling one random observation inside each 0.7-arcdegree (about 78-km) grid, resulting in approximately 4,500 observations for each subsample. Given that the model was run with 100 iterations, this resulted in a total of about 450,000 samples used to build our GS models. Parameter tuning for each model was performed through the grid-search procedure of Google Earth Engine^96^ to explore the results of a suite of machine-learning models trained on the 49 covariates. For each of the models, we ran 48 discrete parameter sets covering the total grid space of 700 possible parameter combinations. Performance of each model was assessed using the coefficient of determination (*R*^2^) values from tenfold cross-validation (Supplementary Table 1) and we retained the best models from each bootstrapped spatial subsample. All *R*^2^ values reported throughout the manuscript represent the coefficient of determination relative to the 1:1 line of observed versus predicted values, which is equivalent to a standardized mean squared error.

As an alternative to testing whether spatial autocorrelation in model residuals affects model-validation statistics, we applied spatially buffered LOO-CV using the respective autocorrelation distances as buffer radii (Supplementary Table 1). In this procedure, each data point is predicted by a model that uses all data outside the buffer radius of the respective data point as training data. To run the LOO-CV, we used the hyperparameter settings of the best-performing random-forest model based on random tenfold cross-validation.

To create the final maps of current tree carbon density, we used an ensemble approach, whereby we averaged the global predictions from the 100 best random-forest models. By taking the average prediction across several models, ensemble methods minimize the influence of any single prediction, thereby stabilizing variation and minimizing bias that can otherwise arise from extrapolation or overfitting when using a single machine-learning model^99^. Geospatial mapping was also performed in Google Earth Engine^96^.

To account for tree carbon stored belowground as roots, we multiplied our aboveground tree carbon predictions by the pixel-level means or the upper and lower confidence bounds of the proportional contribution of root carbon, using a spatially explicit map of tree root mass fraction^24^ (Supplementary Fig. 16). This map was derived from random-forest models based on 5,170 spatially explicit observations of tree biomass ratios between roots and shoots, covering all continents except Antarctica. Confidence ranges of the pixel-level root mass fraction estimates were based on sampling uncertainty, using a stratified bootstrapping procedure (see methods in ref. ^24^ for details).

To generate the final ground-sourced map of existing total living tree carbon (aboveground and belowground biomass in t C ha^−1^) at 30-arcsec resolution (about 1 km^2^), the total carbon stored at present in living trees (*C*_existing_) was then calculated as:

4 $${C}_{{\rm{existing}}}=\mathop{\sum }\limits_{p=1}^{m}\left({D}_{{\rm{existing}}}\times {{\rm{Area}}}_{{\rm{Pixel}}}\right)$$

in which *D*_existing_ is the living tree carbon density in each pixel and Area_Pixel_ is the area of each pixel.

To evaluate the extent of model interpolation versus extrapolation, that is, how well our training data represent the full multivariate environmental covariate space, we performed an approach based on principal component analysis (PCA)^100^. To do so, we performed PCA on the 49 covariates represented in our training data, using the centring values, scaling values and eigenvectors to transform the 49 covariates into the same PCA spaces. Then we created convex hulls for each of the bivariate combinations from the top 19 principal components (which collectively covered more than 90% of the sample-space variation). Using the coordinates of these convex hulls, we classified whether each pixel falls within or outside each of these convex hulls. In total, 92% of the potential canopy cover area fell within ≥95% of the 171 PCA convex hull spaces computed from our training data (representing the range of environmental conditions in our training data), with most of the outliers existing in arid regions (Supplementary Fig. 17a).

We also tested how well the training data span the variation in the eight human-disturbance layers. In total, 90% of the potential canopy cover area fell within ≥95% of the ten PCA convex hull spaces computed from our training data (Supplementary Fig. 17b).

#### Satellite-derived tree carbon density models

To compare and benchmark our ground-sourced tree carbon models against satellite-derived predictions, we used three state-of-the-art products of current aboveground forest biomass: (1) the latest ESA-CCI forest biomass map published in 2022 by the European Space Agency’s Climate Change Initiative^7,101^; (2) a woody carbon stock map published in 2022 by Walker et al.^2^; and (3) a harmonized woody carbon stock map published in 2020 by Spawn et al.^8^.

The ESA-CCI map represents aboveground living tree biomass for the year 2010 and was produced using satellite data from ALOS-2/PALSAR-2 and a physical-based inversion model that estimates biomass from growing stock volume, wood density and biomass expansion factors, with bias adjustment following the validation framework in ref. ^7^. The map was averaged from 100-m to 1-km^2^ spatial resolution to match the resolution of the covariates. The 1-km^2^ ESA-CCI map was assessed following the validation framework in ref. ^7^, wherein map bias is predicted using a model-based approach based on global reference data. This step reduces mapping bias in areas with statistically significant prediction bias and particularly reduces the underestimation of biomass at high-biomass forests >350 t ha^−1^. The map comes with an uncertainty layer that accounts for spatially correlated errors during spatial averaging. To convert the living tree biomass estimates to carbon, we multiplied tree biomass with biome-specific wood carbon concentrations^77^ (see Supplementary Table 5).

The Walker et al.^2^ map represents woody aboveground carbon stocks for the year 2016 and was created by combining field measurements using airborne and spaceborne (NASA ICESat Geoscience Laser Altimeter System; GLAS) lidar data to yield spatially explicit estimates of aboveground biomass density at the GLAS footprint (about 60-m diameter) scale. Regression models were then used to relate the GLAS-based estimates of aboveground biomass to satellite imagery by the Moderate Resolution Imaging Spectroradiometer (MODIS), ultimately allowing to generate spatially explicit estimates of global aboveground biomass density at a resolution of approximately 500 m. The map was aggregated from 500-m to 1-km^2^ spatial resolution to match the resolution of the covariates and came with an uncertainty layer that accounts for the spatially modelled error, representing the 95% quantile intervals generated by quantile regression forests^2^.

The harmonized map^8^ represents aboveground woody carbon stocks for the year 2010 and was based on the GlobBiomass^102^ map and refined using remotely sensed data for Africa^103^ (see ref. ^8^ for details). The map was aggregated from 300-m to 1-km^2^ spatial resolution to match the resolution of the covariates and came with an uncertainty layer that represents the uncertainty associated with the harmonization correction^8^.

### Geospatial modelling of tree carbon potential

To map the tree carbon potential in the hypothetical absence of humans, we developed four data-driven modelling approaches, with two sets of models developed from ground-sourced data (GS1 and GS2) and two from satellite-derived data (SD1 and SD2).

#### GS models

After training and parameterizing the GS model of current tree carbon density using equation (3), we estimated the potential tree carbon density in forests that could exist in the absence of human disturbance by modifying this equation setting human-disturbance variables to zero and replacing existing canopy cover with potential canopy cover (GS1):

5 $${C}_{{\rm{Potential}}}=\mathop{\sum }\limits_{i=1}^{n}{\overrightarrow{\alpha }}_{i}{\mathop{{\rm{V}}{\rm{a}}{\rm{r}}}\limits^{\longrightarrow }}_{i}^{{\rm{Env}}}+\mathop{\sum }\limits_{j=1}^{m}{\overrightarrow{\beta }}_{j}{\mathop{{\rm{V}}{\rm{a}}{\rm{r}}}\limits^{\longrightarrow }}_{j}^{{\rm{zeroHuman}}}+\overrightarrow{\gamma }{\mathop{{\rm{V}}{\rm{a}}{\rm{r}}}\limits^{\longrightarrow }}^{{\rm{CanopyCover}}}$$

in which ${\mathop{{\rm{V}}{\rm{a}}{\rm{r}}}\limits^{\longrightarrow }}^{{\rm{Env}}}$ are the environmental variables, ${\mathop{{\rm{V}}{\rm{a}}{\rm{r}}}\limits^{\longrightarrow }}^{{\rm{zeroHuman}}}$ are the scaled human-disturbance variables set to zero and ${\mathop{{\rm{V}}{\rm{a}}{\rm{r}}}\limits^{\longrightarrow }}^{{\rm{CanopyCover}}}$ is the current canopy cover^95^, which was replaced by potential canopy cover^3^ after model training for the prediction of the total carbon potential. This allowed us to train the model including information on current (2010) forest canopy cover^95^ and then to predict the tree carbon potential inside the potential canopy cover by replacing current canopy cover with the ‘natural’ canopy cover expected in the absence of humans^3^.

For the second GS model of potential tree carbon density (GS2), we included only data from regions with minimal human disturbance and used the 40 environmental covariates and canopy cover as predictors:

6 $${C}_{{\rm{Potential}}}=\mathop{\sum }\limits_{i=1}^{n}{\overrightarrow{\alpha }}_{i}{\mathop{{\rm{V}}{\rm{a}}{\rm{r}}}\limits^{\longrightarrow }}_{i}^{{\rm{Env}}}+\overrightarrow{\gamma }{\mathop{{\rm{V}}{\rm{a}}{\rm{r}}}\limits^{\longrightarrow }}^{{\rm{CanopyCover}}}$$

in which ${\mathop{{\rm{V}}{\rm{a}}{\rm{r}}}\limits^{\longrightarrow }}^{{\rm{Env}}}$ are the environmental variables and ${\mathop{{\rm{V}}{\rm{a}}{\rm{r}}}\limits^{\longrightarrow }}^{{\rm{CanopyCover}}}$ is the current canopy cover^95^, which was replaced by potential canopy cover^3^ after model training.

The GS2 model differs from the GS1 model in a reduced number of observations (only pixels with minimal human disturbance) and a reduced number of predictors (no human-disturbance variables). Regions with minimal human disturbance were defined as pixels located in: (1) a protected area, that is, strict nature reserve or wilderness area^94^; (2) intact forest, that is, contiguous forest with no remotely detected signs of human activity and a minimum area of 500 km^2^ (ref. ^26^); and/or (3) pixels in which human modification is <1% following ref. ^91^. To minimize the influence of uneven distribution of observations and spatial autocorrelation on model training, we applied bootstrapped spatial subsampling (100 iterations), similar to the GS1 models, whereby—for each subsample—we randomly sampled one observation in each 0.25 arcdegree, which resulted in about 4,500 observations for each subsample.

As for the predictions of current tree carbon, for both the GS1 and GS2 models, we added root carbon^24^ to generate maps representing total living tree carbon potential in the absence of human disturbance.

#### SD models

The two types of SD model were run with the ESA-CCI^18^, Walker et al.^2^ and harmonized maps^8^ of current woody carbon as input data, resulting in six model combinations (two model types and three input datasets). As for the GS1 model, model structure and parameterization of the first SD model of potential living tree carbon (SD1) followed equation (5). Similarly, as for the GS2 model, the second SD model of potential tree carbon density (SD2) followed equation (6), and we trained the model using only biomass density information from areas with minimal human disturbance inside protected areas (strict nature reserve or wilderness area)^94^ and/or intact forest landscapes^26^.

For both the SD1 and SD2 models, we conducted a bootstrap subsampling approach similar to the GS models, whereby about 4,500 sample points were drawn 100 times with replacement. For the SD1 model, observations were drawn randomly, given that the models were built from global maps for which data are distributed equally across all global forest areas. For the SD2 model, we applied spatial subsampling, randomly sampling one observation in each 1-arcdegree grid to account for the uneven distribution of areas with minimal human disturbance across the globe. For each subsample, we ran 48 discrete parameter sets covering the total grid space of 700 possible parameter combinations and kept the parameter set with the highest coefficient of determination (*R*^2^) based on tenfold cross-validation. To obtain the final predictions, we averaged the predictions from the 100 random-forest models.

To test for spatial autocorrelation in model residuals, we calculated the Moran’s *I* index of the residuals from generalized additive models at different spatial scales (0–1,000 km) and, for each model, found spatial autocorrelation at distances of up to 550–900 km (Supplementary Fig. 15e–j). To test for the effect of spatial autocorrelation on model validation statistics, we then ran LOO-CV models for each of the 100 bootstrapped subsamples, using the respective autocorrelation distances as buffer radii and the hyperparameter settings of the best-performing random-forest model based on random tenfold cross-validation (Supplementary Table 1).

### Adding dead wood, litter and soil carbon to scale living tree carbon to total ecosystem carbon

#### Dead wood and litter biomass

To account for forest carbon stored in dead wood and litter, we obtained forest-type-level carbon ratios from previous studies^19,104^. Means and confidence ranges of the ratios between dead wood and litter carbon and living tree carbon for tropical, temperate and boreal forests were calculated from forest-type estimates of total living biomass, dead wood and litter from Table S3 in ref. ^19^. Means and confidence ranges for dryland forests were calculated from Table 1 in ref. ^104^, using all sites for which data on plant aboveground and belowground biomass and litter was available. The ratios between dead wood and litter carbon and living tree carbon were 22% (95% confidence range = 15–33%), 33% (30–37%), 80% (68–94%) and 21% (2–40%) for tropical, temperate, boreal and dryland forests, respectively. We then multiplied pixel-level living tree carbon values by these percentages to estimate the means and confidence bounds of dead wood and litter carbon for each pixel (Table 1).

#### Soil carbon

Using the soil potential map ref. ^23^, which represents the effects of anthropogenic land-use and land-cover changes on soil organic carbon in the top 2 m (ref. ^23^) over the past 12,000 years, we extracted estimates of soil carbon potential in the absence of humans (difference between soil carbon 10,000 BC and current soil carbon) for all pixels that would naturally support trees (potential canopy cover^3^ ≥ 10%; Table 1). Associated spatial-prediction uncertainties (absolute errors) were calculated by fitting a spatial-prediction model to the prediction residuals of the cross-validated original model and applying this error model over the whole area of interest^23^.

### Model uncertainty

For each of the GS and SD models, the 100 bootstrapped models of aboveground tree carbon potential were used to calculate per-pixel coefficient-of-variation values (standard deviation divided by the mean predicted value) as a measure of sampling uncertainty (hereafter referred to as bootstrap prediction uncertainty; Supplementary Figs. 1 and 2). Using the bootstrapped models, we also calculated 95% confidence ranges of estimates, allowing us to represent uncertainty ranges for each aboveground carbon model. To represent the uncertainty in canopy cover of the forest inventory plots, we ran the GS1 and GS2 models for both the upper and lower canopy cover estimates. To represent data uncertainty of the SD models, we ran the SD1 and SD2 models using three different input datasets (ESA-CCI^18^, Walker et al.^2^ and harmonized biomass maps^8^). Uncertainty in belowground tree carbon was derived by multiplying the upper and lower confidence ranges of aboveground tree carbon values with the upper and lower confidence ranges of spatially explicit root mass fractions^24^, thus representing uncertainties in both root mass fraction and aboveground biomass. Using the entire confidence range of total (aboveground and belowground) living tree carbon, including sampling and data uncertainty, we then calculated the uncertainty in dead wood and litter biomass by multiplying the upper and lower confidence ranges of total living tree carbon values with the upper and lower confidence ranges of the forest-type-specific ratios between dead wood and litter carbon and living tree carbon (see ‘Dead wood and litter biomass’ section). Dead wood and litter biomass uncertainty was thus the result of uncertainties in both dead wood and litter-to-tree biomass ratios and tree biomass. Spatially explicit uncertainties in soil carbon potential were derived from maps of absolute errors in organic carbon density at 0–200 cm soil depth provided in ref. ^23^. Propagation of uncertainty was done by summing all individual uncertainties and assuming that they are uncorrelated.

To quantify the relative contribution of the different sources of uncertainty to the overall uncertainty in our models, we divided the absolute uncertainty of each uncertainty type by the sum of all uncertainties (Fig. 4). This partitioning allows for relative comparison in uncertainty among sources, but otherwise does not necessarily reflect total model uncertainty owing to overlap and correlation across sources of uncertainty.

### Carbon potential partitioning

On the basis of our carbon models, we could generate estimates of (1) the relative contribution of forest degradation (that is, reduced tree carbon within the existing canopy cover) to the difference between current and potential carbon stocks (hereafter referred to as conservation potential) and (2) the relative contribution of deforestation (that is, declines in canopy cover owing to land-use change in areas that would naturally support trees) to the difference between current and potential carbon stocks (hereafter referred to as restoration potential). Specifically, to estimate the relative contribution of forest degradation (conservation potential) and deforestation (restoration potential) to the difference between current and potential carbon stocks, we first attributed the proportional amount of the extra carbon predicted by our model to the extra canopy cover expected in the absence of humans. For example, for a pixel in which potential canopy cover is twice as high as current canopy cover and for which the predicted potential carbon is also twice as high as current carbon, the extra carbon is attributed only to the difference in canopy cover (restoration potential). For pixels in which the potential increase in tree carbon exceeded the proportional increase in canopy cover, the carbon potential fraction exceeding the proportional increase in canopy cover was equally distributed across the total potential canopy cover of the pixel. For pixels in which potential canopy cover was the same as current canopy cover, we attributed the difference between current and potential tree carbon stocks to forest degradation (conservation potential).

Throughout the text, we refer to conservation potential as the difference between current and potential carbon in existing forests, which was computed by subtracting the carbon stored at present inside existing forests from the expected carbon in these forests in the absence of human disturbance. We refer to restoration potential as the difference between current and potential carbon outside existing forests, which was estimated as the expected carbon in non-forest areas that would naturally support trees in the absence of human disturbances^3^. Finally, the total difference between current and potential carbon refers to the sum of the conservation and restoration potentials (Figs. 3 and 5).

To estimate the existing and potential carbon within biomes (Supplementary Table 2), forest classes (tropical, temperate, boreal and dryland; Supplementary Table 3) and countries (Fig. 5e), we used the World Wide Fund for Nature (WWF) biome definitions^71^ and country boundaries from the world boundary map^105^. Forests were classified into four broad categories (tropical, temperate, boreal and dryland)^71^. Tropical forest includes six biomes: tropical and subtropical moist broadleaf forest, tropical and subtropical dry broadleaf forest, tropical and subtropical coniferous forest, tropical and subtropical grassland, savannah and shrubland, flooded grassland and savannah, and mangroves; temperate forest includes four biomes: temperate broadleaf and mixed forest, conifer forest, temperate grassland, savannah and shrubland, and montane grassland and shrubland; boreal forest includes two biomes: boreal forest/taiga and tundra; dryland refers to the two biomes Mediterranean forest, woodland and scrub, and desert and xeric shrubland.

To partition potential carbon stocks into different land-cover types, we integrated four land-cover maps^88–90,106^, providing information on the relative area of a pixel that is covered by urban area, cropland, permanent pasture, rangeland, urban area, forest, water body and ice and snow. The difference between current and potential tree carbon stocks predicted by our models was then allocated to the land-cover types urban area, cropland, permanent pasture, rangeland, urban area and forest in proportion to their relative pixel coverage. Low-human-pressure land was defined as the proportion of a non-forest pixel (<10% canopy cover) that could not be attributed to pasture, rangeland, cropland, urban area, water body or ice and snow. All areas in forest pixels that could not be attributed to pasture, rangeland, cropland, urban area, water body or ice and snow were attributed to forest. Global information on forest plantations came from ref. ^28^ and we only considered plantations if they covered more than 10% of the canopy area in a pixel.

### Meta-analysis of previous studies on the global carbon potential

To gain insight into the forest carbon potential estimated by previous studies, we reviewed publications that applied diverse approaches to quantify the potential carbon storage capacity of global forests. These studies fall into two types of estimate. The first type included studies reporting the total carbon that could be stored in global forests in the absence of human activities (Fig. 3b). The second type encompassed studies reporting the extra potential carbon that could be stored in the global forests, that is, the difference between current and potential carbon stocks (Fig. 3d). In total, we found 20 estimates of the total carbon potential and nine estimates of the difference between current and potential carbon stocks. These estimates were derived from four different approaches: inventory-based empirical estimates, mechanistic models, ensemble models and data-driven models. Inventory-based estimates comprise studies that estimated the global carbon potential from maximum forest carbon densities observed in climate zones or ecoregions based on inventory data^1,55,58^. Mechanistic-model estimates included studies that used mechanistic models, such as Earth system models, to estimate the carbon potential of global forests^64,67^. Ensemble-model estimates consisted of studies that used a variety of existing biomass maps to estimate the global carbon potential from maximum forest carbon densities in climate zones or ecoregions^4^. Last, the data-driven model category encompassed studies that used extensive global carbon density observations to train global models based on environmental covariates^2^. References to the studies included in this meta-analysis are shown in the legend of Fig. 3 and Supplementary Table 7.

All analyses were conducted in Google Earth Engine^96^ and R (v. 3.6.3)^107^. All figures were created in R (v. 3.6.3)^107^.

## Online content

Any methods, additional references, Nature Portfolio reporting summaries, source data, extended data, supplementary information, acknowledgements, peer review information; details of author contributions and competing interests; and statements of data and code availability are available at 10.1038/s41586-023-06723-z.

### Supplementary information

Supplementary Information Supplementary Figs. 1–17, Supplementary Tables 1–7 and Supplementary References.

## Data Availability

Data and code are available at GitHub: 10.5281/zenodo.10021968.
