# Supplementary material for: Integrated global assessment of the natural forest carbon potential
Source: Nature. 2023 Nov 13;624(7990):92–101. doi: 10.1038/s41586-023-06723-z (PMC10700142; doi:10.1038/s41586-023-06723-z)
Supplement: Supplementary file 1 — Supplementary Figs. 1–17, Supplementary Tables 1–7 and Supplementary References. [file 41586_2023_6723_MOESM1_ESM.docx]

**Integrated global assessment of the natural forest carbon potential**

Lidong Mo and Constantin M. Zohner et al.

**This PDF file includes:**

Supplementary Figures S1-S17

Supplementary Table S1-S7

Supplementary references

| **Page** | **Supplementary Figures/Tables** |
| --- | --- |
| 1 | **Figure S1. Prediction uncertainty of the ground-sourced (GS; a-d) and satellite-derived (SD; e-j) models of tree carbon potential.** |
| 2 | **Figure S2. Prediction uncertainty of the two ground-sourced models of existing tree carbon.** |
| 3 | **Figure S3**. **Performance and validation of the ground-sourced (a-d) and satellite-derived models (e-j)**. |
| 4 | **Figure S4. Model performance and corresponding sample size of the type 1 models along the human modification gradient**. |
| 5 | **Figure S5. Global maps of present tree carbon based on the ground-sourced (GS) and satellite-derived (SD) models.** |
| 6 | **Figure S6.** **Comparison of present live tree carbon along latitude between the two ground-sourced (GS) and three satellite-derived (SD) estimates.** |
| 7 | **Figure S7. Comparison of aboveground tree carbon predictions between satellite-derived products and our ground-sourced model.** |
| 8 | **Figure S8. The total live tree carbon potential in the absence of humans for the ground-sourced (GS) and satellite-derived (SD) models.** |
| 9 | **Figure S9. Comparison of the live tree carbon potential estimated from the ground-sourced (x axis) and satellite-derived (y axis) models**. |
| 10 | **Figure S10. Difference between present and potential total ecosystem carbon within forest areas (a), low human pressure land outside forest areas (b), pasture land (c), rangeland (d), cropland (e), and urban areas (f).** |
| 11 | **Figure S11. Plot area per biome covered by the ground-sourced forest inventory data.** |
| 12 | **Figure S12. DBH-based allometric models of tree biomass for extratropical biomes obtained from the GlobAllomeTree database**^8^ **and following the methodology of Jenkins et al.** |
| 13 | **Figure S13. Histograms of observed tree carbon densities (log-transformed) in each biome based on the ground-sourced GFBi data.** |
| 14 | **Figure S14. Variable importance of 10 environmental metrics based on random forest MDA values for the ground-soured (*Upper/Lower canopy cover*) and satellite-derived (*Harmonized, ESA-CCI, Walker et al.*) models of tree carbon.** |
| 15 | **Figure S15. Residual spatial autocorrelation (Moran’s I) of the ground-sourced (a-d) and satellite-derived (e-j) models, assessed using generalized additive models along a distance gradient.** |
| 16 | **Figure S16. Map showing the ratio between root and shoot biomass for global forest areas**. |
| 17 | **Figure S17. Representation of the training data for the ground-sourced model considering all covariates (a) or only the human disturbance covariates (b).** |
| 18 | **Table S1. The global performance of the ground-sourced (SD) and satellite-derived (SD) models.** |
| 19 | **Table S2. Live tree carbon potential within biomes.** |
| 20 | **Table S3. Live tree carbon stocks in the world’s forests.** |
| 21 | **Table S4. Biome-level allometric equations of the non-tropical biomes from the GlobAllomeTree database**^8^ **and following the methodology of Jenkins et al.** |
| 22 | **Table S5. Biome-level carbon concentrations of woody biomass based on Martin et al.** |
| 23 | **Table S6. List of the 49 covariates used in the models of carbon potential.** |
| 25 | **Table S7. Estimates of the global living tree carbon potential from previous studies.** |
| 26 | **References** |


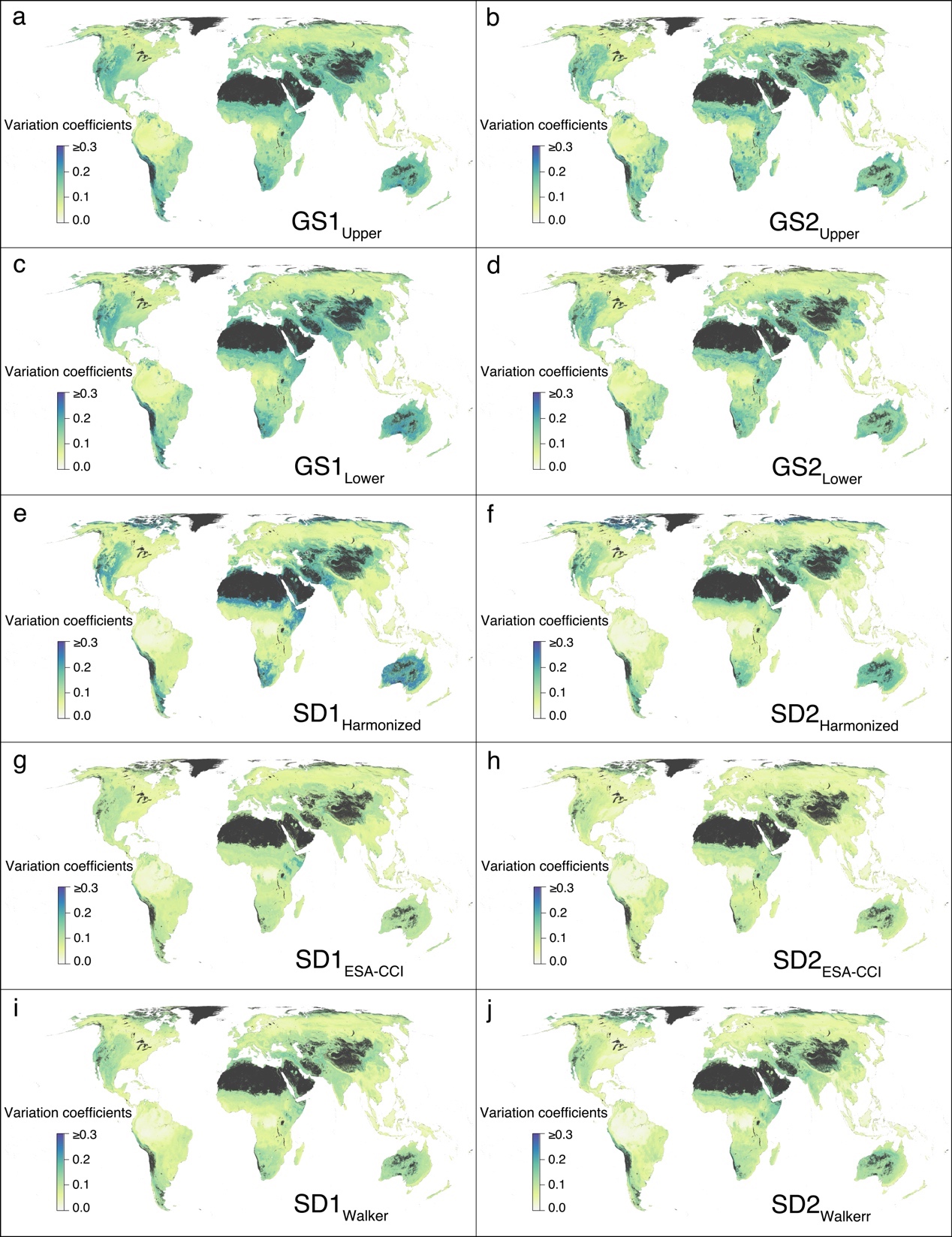


**Figure S1. Prediction uncertainty of the ground-sourced (GS; a-d) and satellite-derived (SD; e-j) models of tree carbon potential.** Values represent bootstrapped coefficients of variation (standard deviation divided by mean) of living tree carbon. Maps are projected at 30 arcsec (~1 km^2^) resolution. **a**, GS1_Upper_ model; **b**, GS2_Upper_ model; **c**, GS1_Lower_ model; **d**, GS2_Lower_ model; **e**, SD1_Harmonized_ model; **f**, SD2_Harmonized_ model; **g**, SD1_ESA-CCI_ model; **h**, SD2_ESA-CCI_ model; **i**, SD1_Walker_ model; and **j**, SD2_Walker_ model.


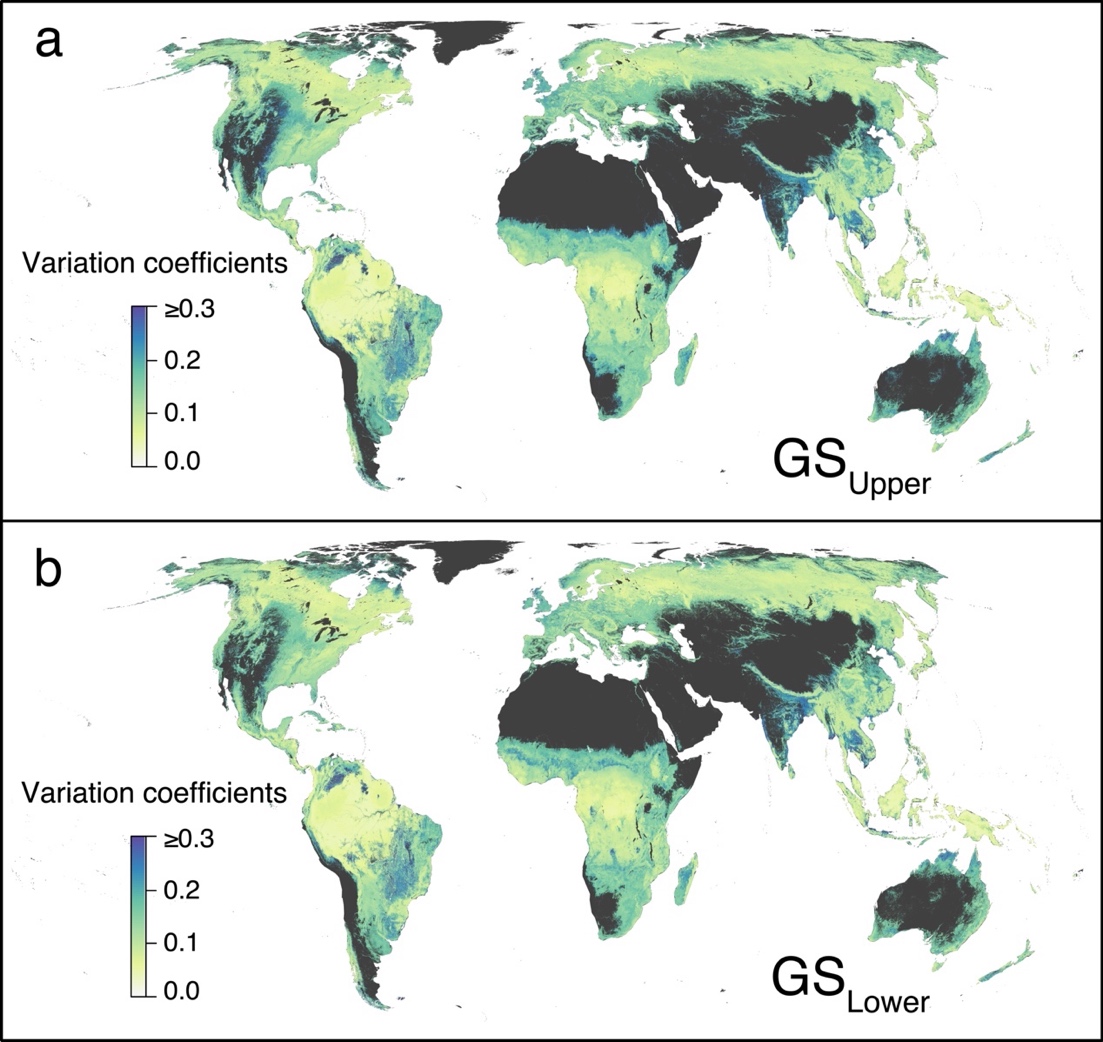


**Figure S2. Prediction uncertainty of the two ground-sourced models of existing tree carbon. a,** Model based on upper canopy cover boundaries (GS_Upper_) to convert carbon per plot to carbon per pixel. **b**, Model based on upper canopy cover boundaries (GS_Lower_). Values represent bootstrapped coefficients of variation (standard deviation divided by mean) of live tree carbon. Maps are projected at 30 arcsec (~1 km^2^) resolution.


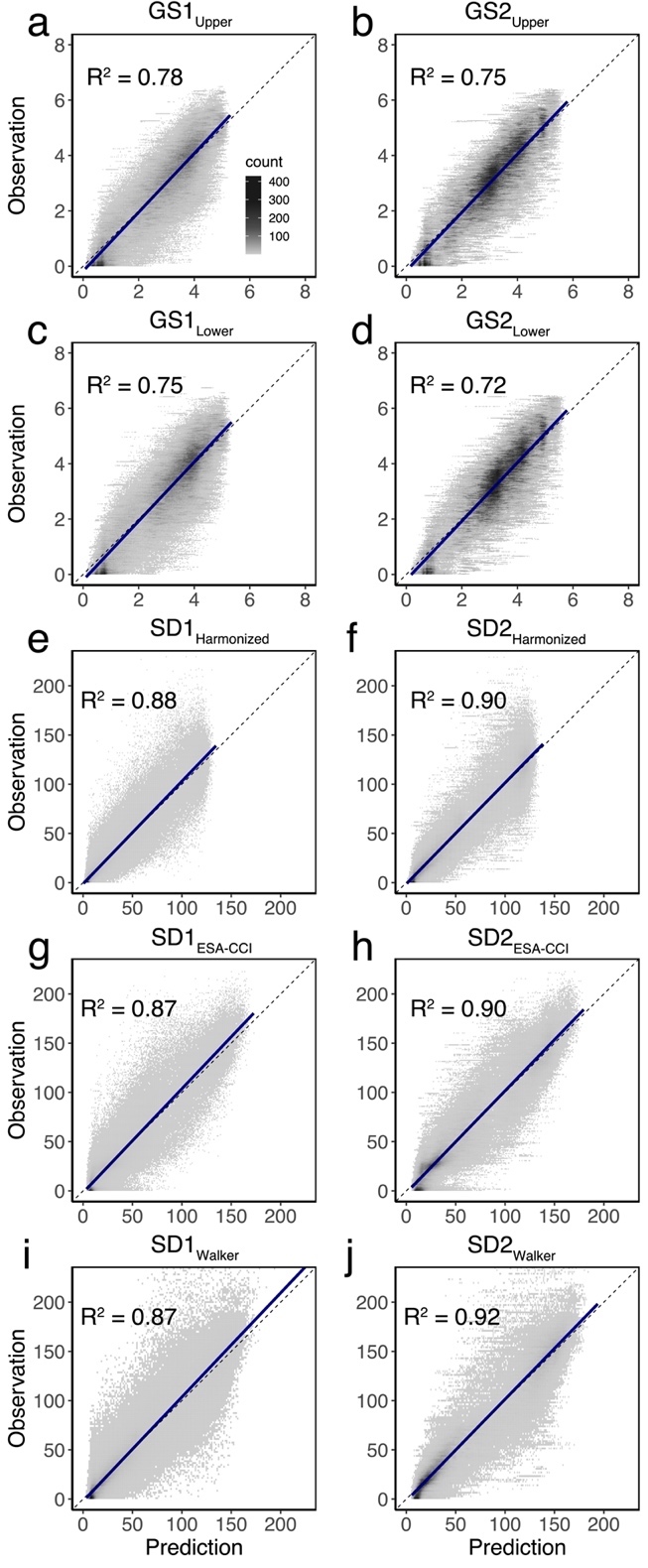


**Figure S3**. **Performance and validation of the ground-sourced (a-d) and satellite-derived models (e-j)**. Each panel presents the relationship between predicted *versus* observed carbon density values for each model based on 10-fold cross validation. The solid black lines indicate the linear regression between predicted and observed values; the dashed line represents the 1:1 line. **a**, GS1_Upper_ model; **b**, GS2_Upper_ model; **c**, GS1_Lower_ model; **d**, GS2_Lower_ model; **e**, SD1_Harmonized_ model; **f**, SD2_Harmonized_ model; **g**, SD1_ESA-CCI_ model; **h**, SD2_ESA-CCI_ model; **i**, SD1_Walker_ model; and **j**, SD2_Walker_ model.


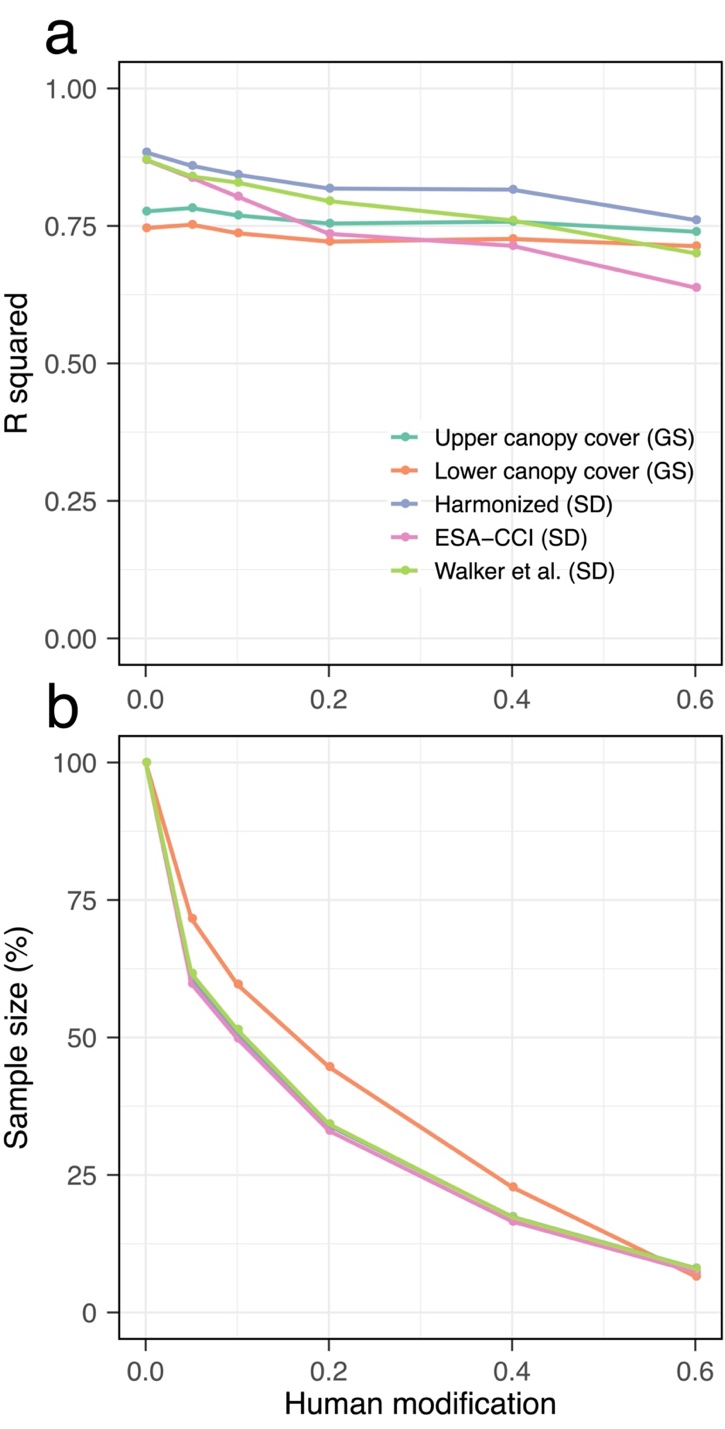


**Figure S4. Model performance and corresponding sample size of the type 1 models along the human modification gradient**. **a**, Model performance of the type 1 models across various levels of human modification intensity using 10-fold cross-validation. For instance, the R^2^ values at 40% human modification represent the predictive accuracy of the models for regions with a human modification >40%. **b**, Sample sizes (as proportion of total observations) used for calculating the model performance within disturbed regions.


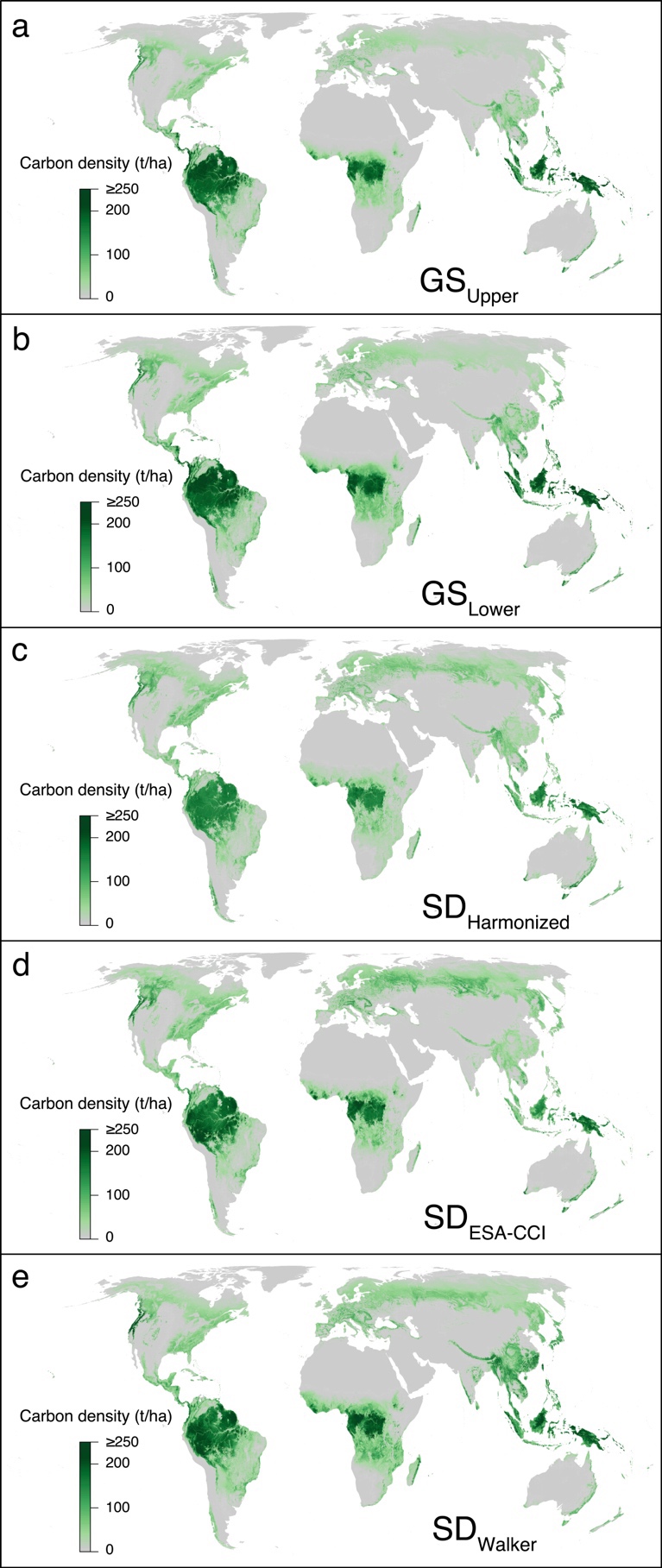


**Figure S5. Global maps of present tree carbon based on the ground-sourced (GS) and satellite-derived (SD) models.** Values represent total (above- and belowground) live tree carbon in tons carbon per hectare. Map projected at 30 arcsec (~1 km^2^) resolution. **a**, GS_Upper_ model; **b**, GS_Lower_ model; **c**, SD_Harmonized_ model; **d**, SD_ESA-CCI_ model; **e**, SD_Walker_ model.


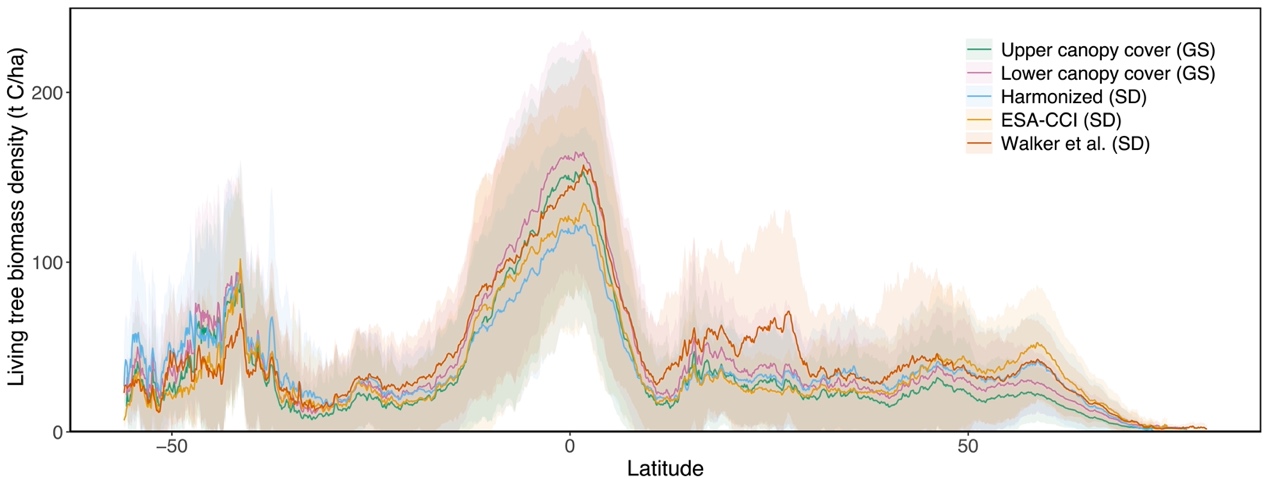


**Figure S6.** **Comparison of present live tree carbon along latitude between the two ground-sourced (GS) and three satellite-derived (SD) estimates.** Mean (± standard deviation) pixel-level tree carbon densities for each 0.1-degree latitude.


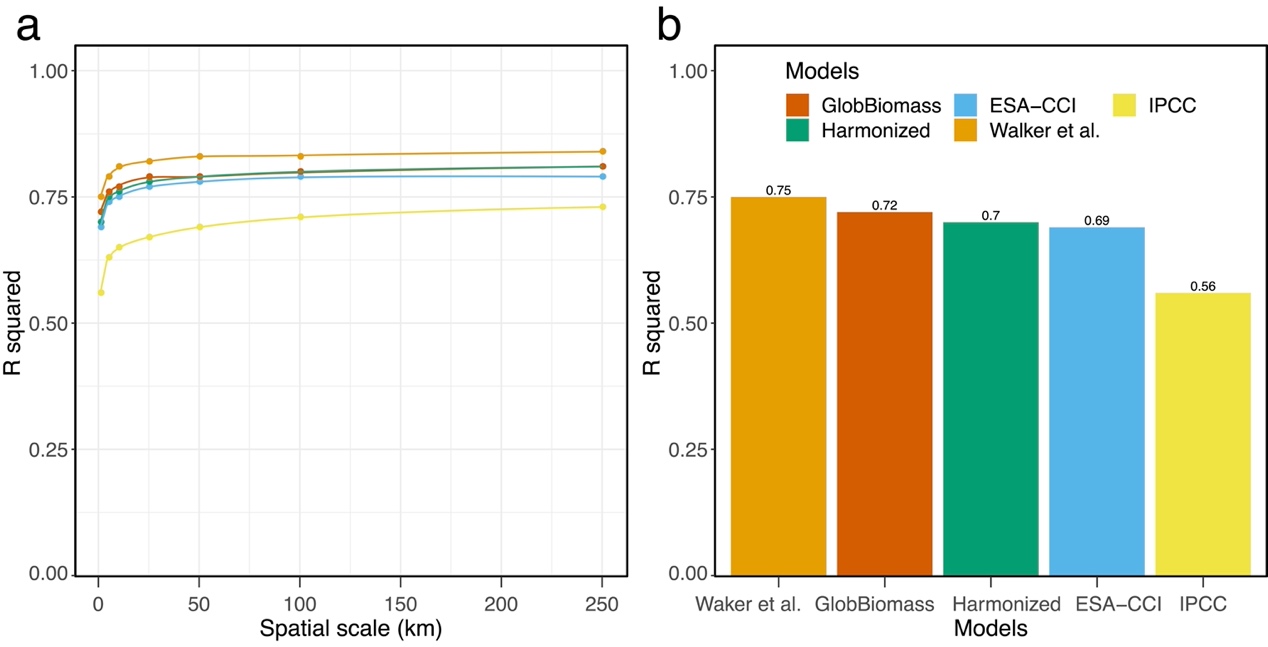


**Figure S7. Comparison of aboveground tree carbon predictions between satellite-derived products and our ground-sourced model. a**, Coefficient of determination values (R^2^s) for the correlation between the Walker et al.^1^, ESA-CCI^2^, GlobBiomass^3^, Harmonized^4^ or IPCC^5^ predictions and our ground-sourced predictions at varying spatial scales, i.e., aggregating the predictions within 1 x 1 km to 250 x 250 km pixels. **b**, R^2^ values between the four satellite-derived models and our ground-sourced model at 1 km^2^ spatial resolution.


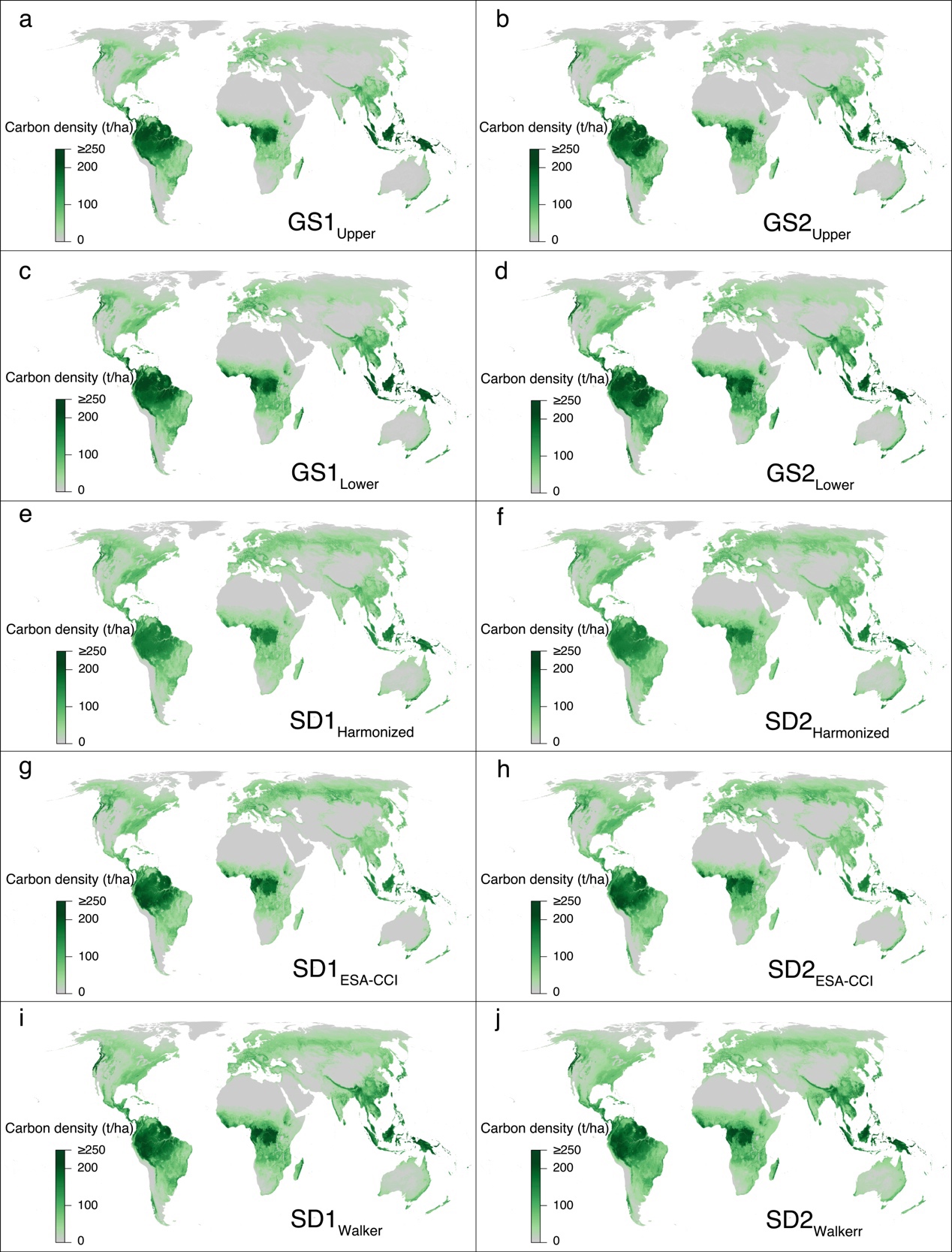


**Figure S8. The total live tree carbon potential in the absence of humans for the ground-sourced (GS) and satellite-derived (SD) models.** The maps represent the potential live tree carbon estimates expected in the absence of humans, within the natural canopy cover area of ~4.4 billion ha^6^. **a**, GS1_Upper_ model; **b**, GS2_Upper_ model; **c**, GS1_Lower_ model; **d**, GS2_Lower_ model; **e**, SD1_Harmonized_ model; **f**, SD2_Harmonized_ model; **g**, SD1_ESA-CCI_ model; **h**, SD2_ESA-CCI_ model; **i**, SD1_Walker_ model; and **j**, SD2_Walker_ model.


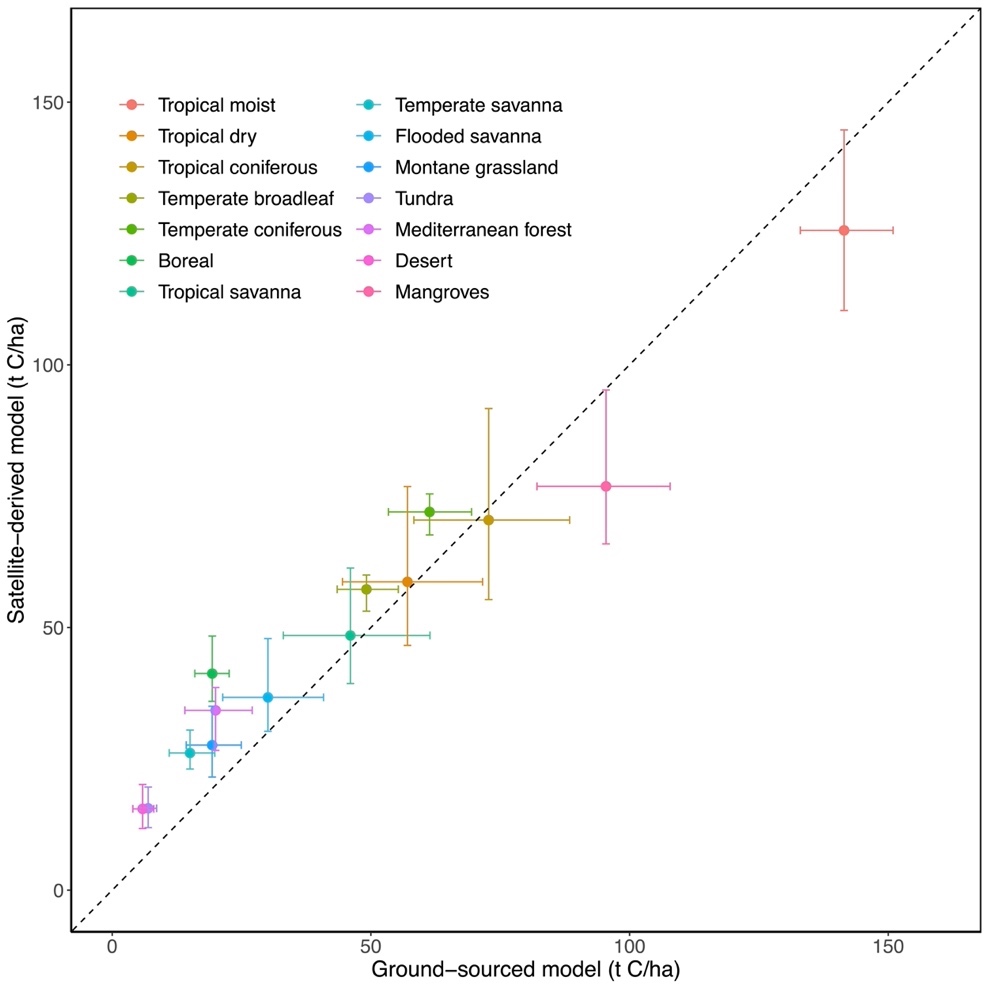


**Figure S9. Comparison of the live tree carbon potential estimated from the ground-sourced (x axis) and satellite-derived (y axis) models**. Each point represents the biome-level average of the live tree carbon potential in tons carbon per hectare. The error bars indicate the corresponding minimum and maximum estimates across the different models based on 95% confidence intervals.


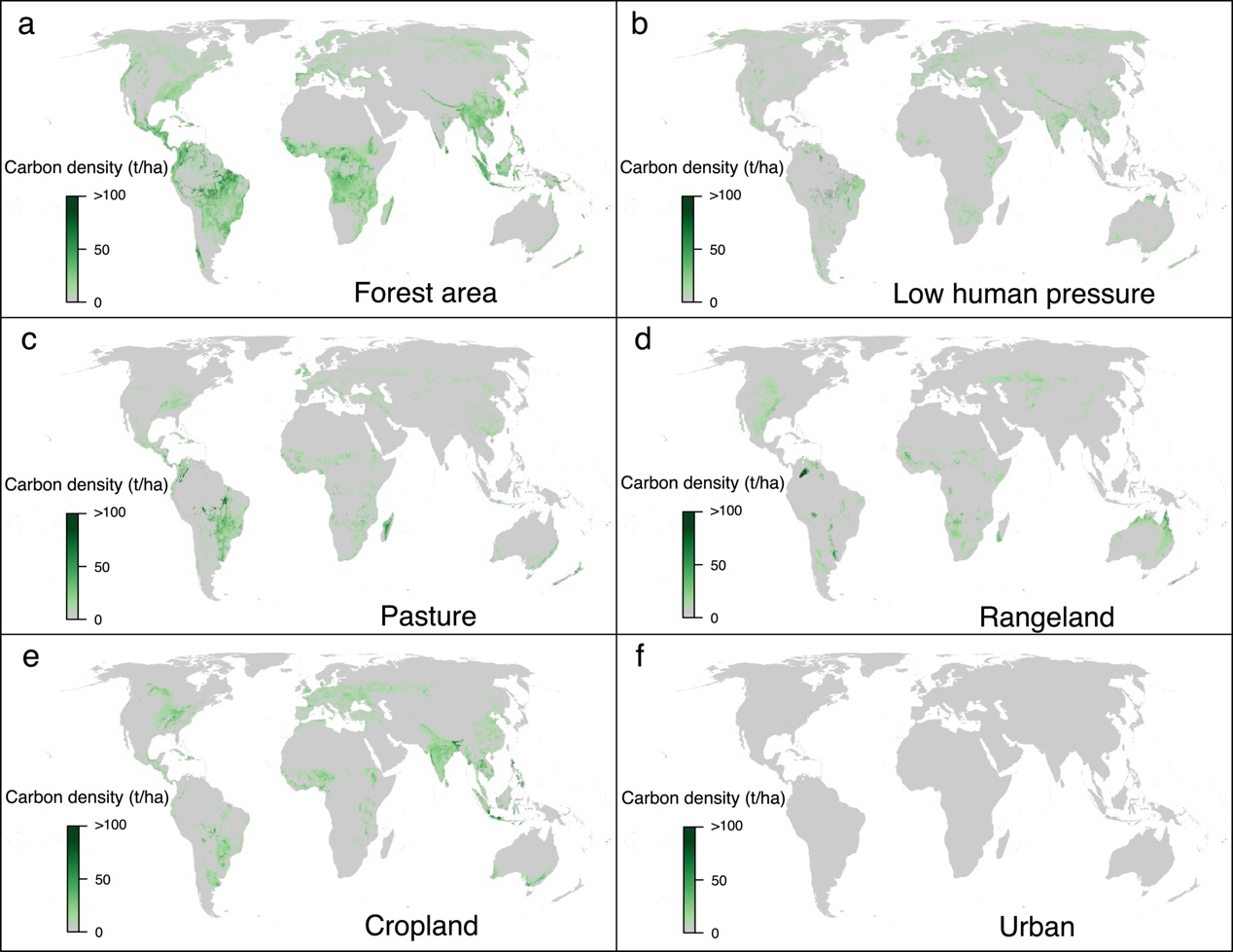


**Figure S10. Difference between present and potential total ecosystem carbon within forest areas (a), low human pressure land outside forest areas (b), pasture land (c), rangeland (d), cropland (e), and urban areas (f).** Values represent above- and belowground live tree carbon in tons carbon per hectare.


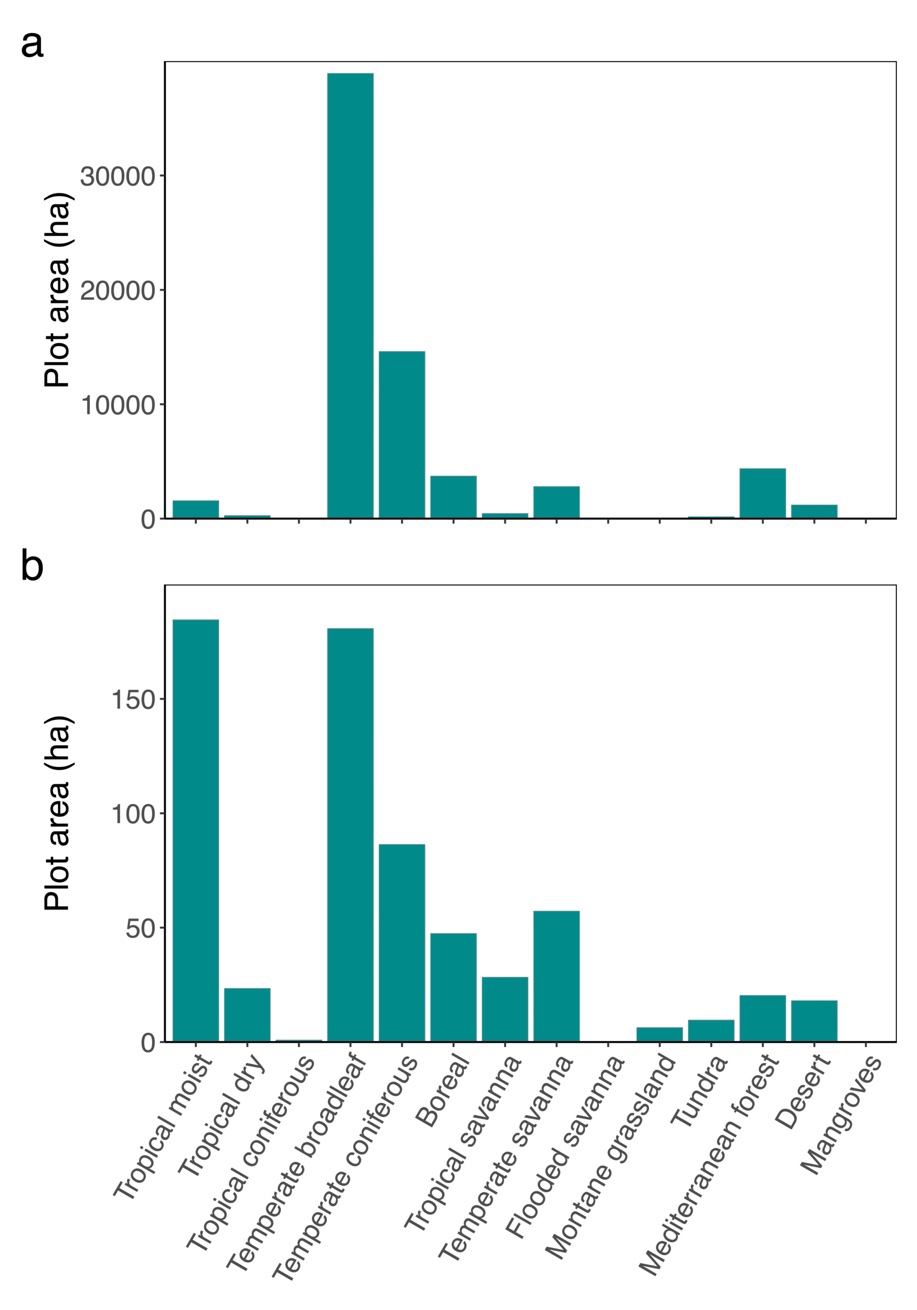


**Figure S11. Plot area per biome covered by the ground-sourced forest inventory data. a,** The biome-level forest plot area covered by the GFBi data. **b**, The biome-level plot area included in each of the 100 bootstrapped subsamples used to model carbon densities.


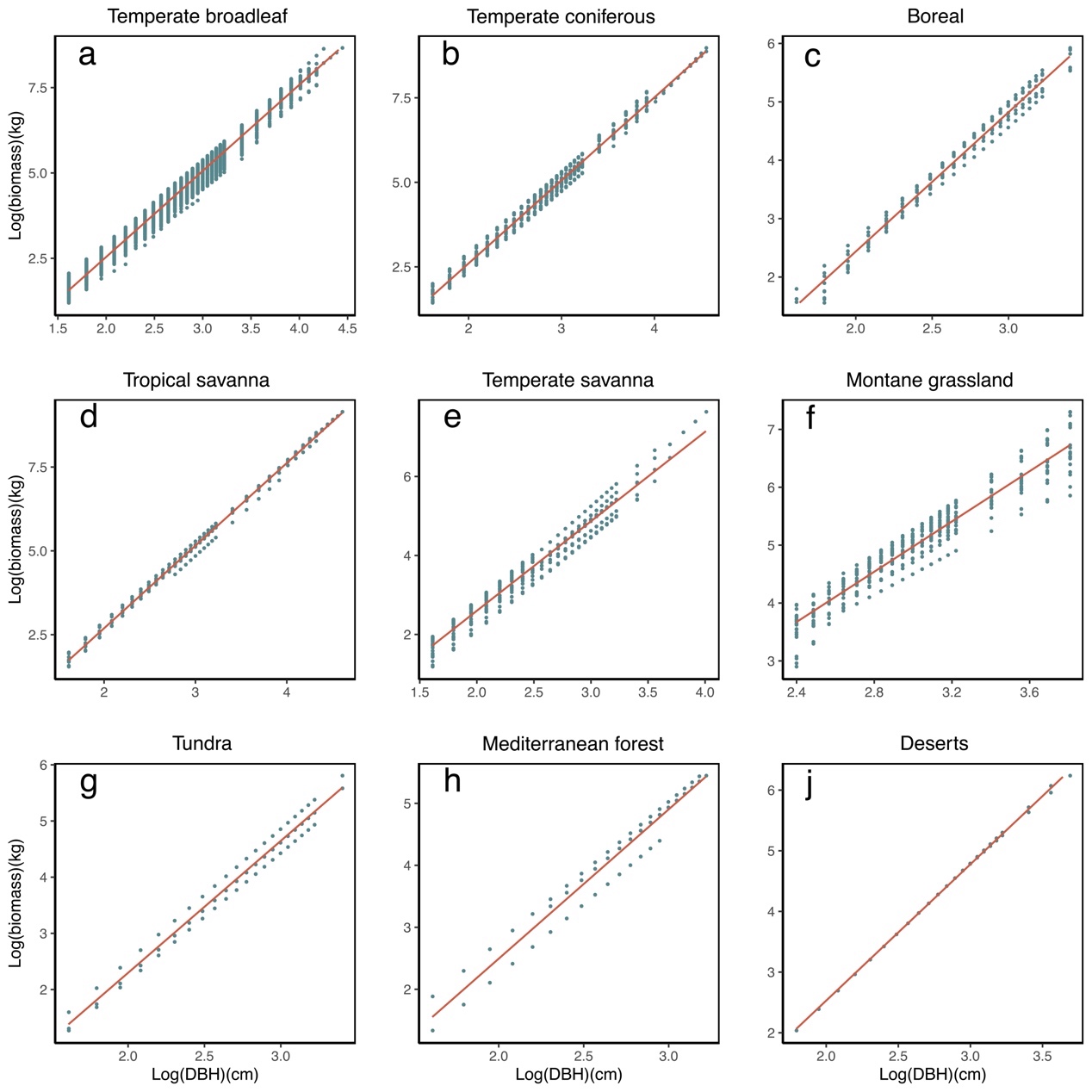


**Figure S12. DBH-based allometric models of tree biomass for extratropical biomes obtained from the GlobAllomeTree database**^8^ **and following the methodology of Jenkins et al.**^9^. For tropical regions, the allometric model of Chave et al.^10^, available through the R package “BIOMASS”^11^, was applied.


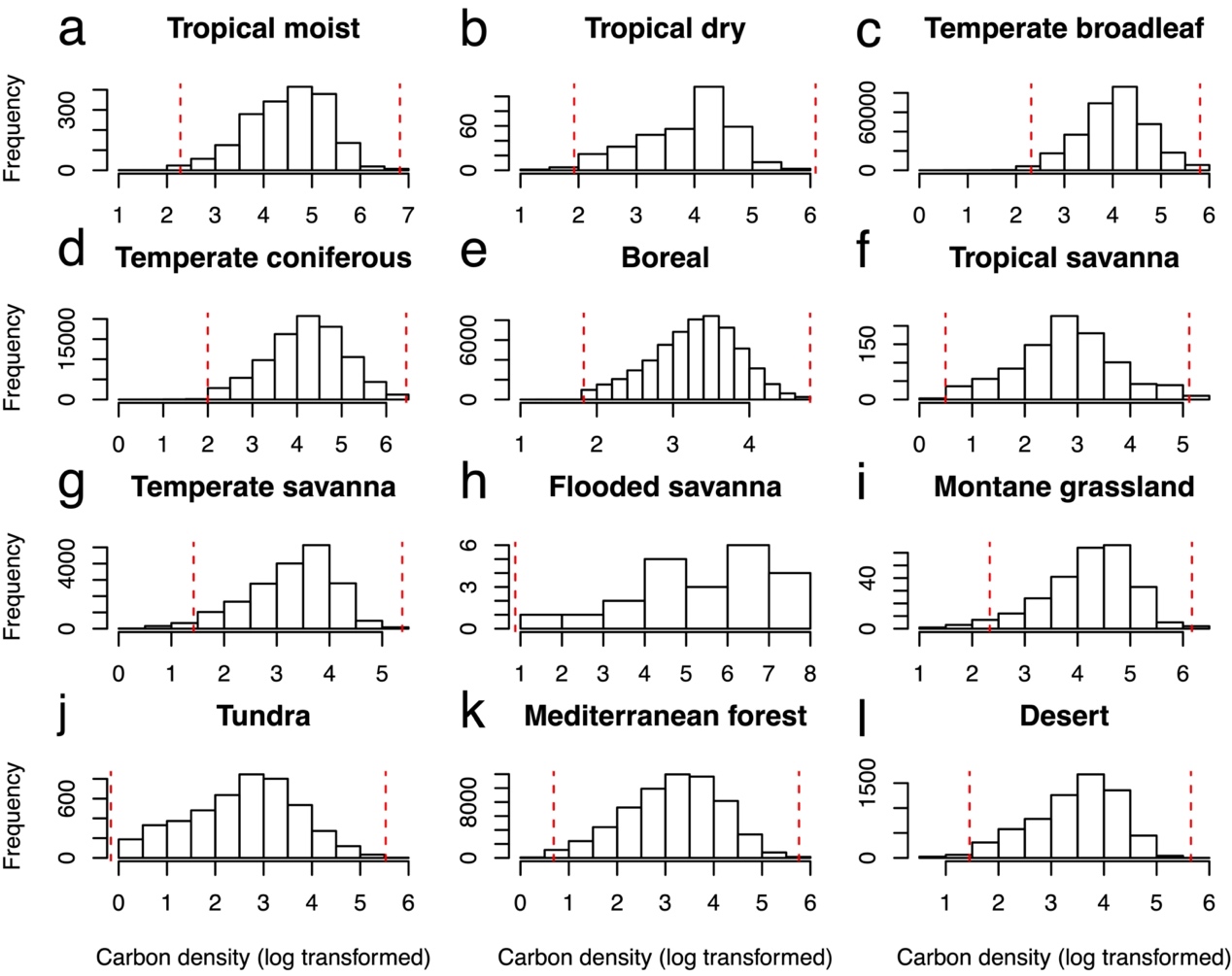


**Figure S13. Histograms of observed tree carbon densities (log-transformed) in each biome based on the ground-sourced GFBi data.** The red dashed lines show the data cleaning cutoffs based on the median absolute deviation (MAD). Left lines indicate the lower boundary (median - 2.5 MAD), right lines indicate the upper boundary (median + 2.5 MAD). Observations that fell between the two lines were used for the analyses. Small outlier values (< median - 2.5 MAD) were kept if they fell in human-modified non-forest landscapes, i.e., regions with a human disturbance index > 10% and canopy cover < 10%.


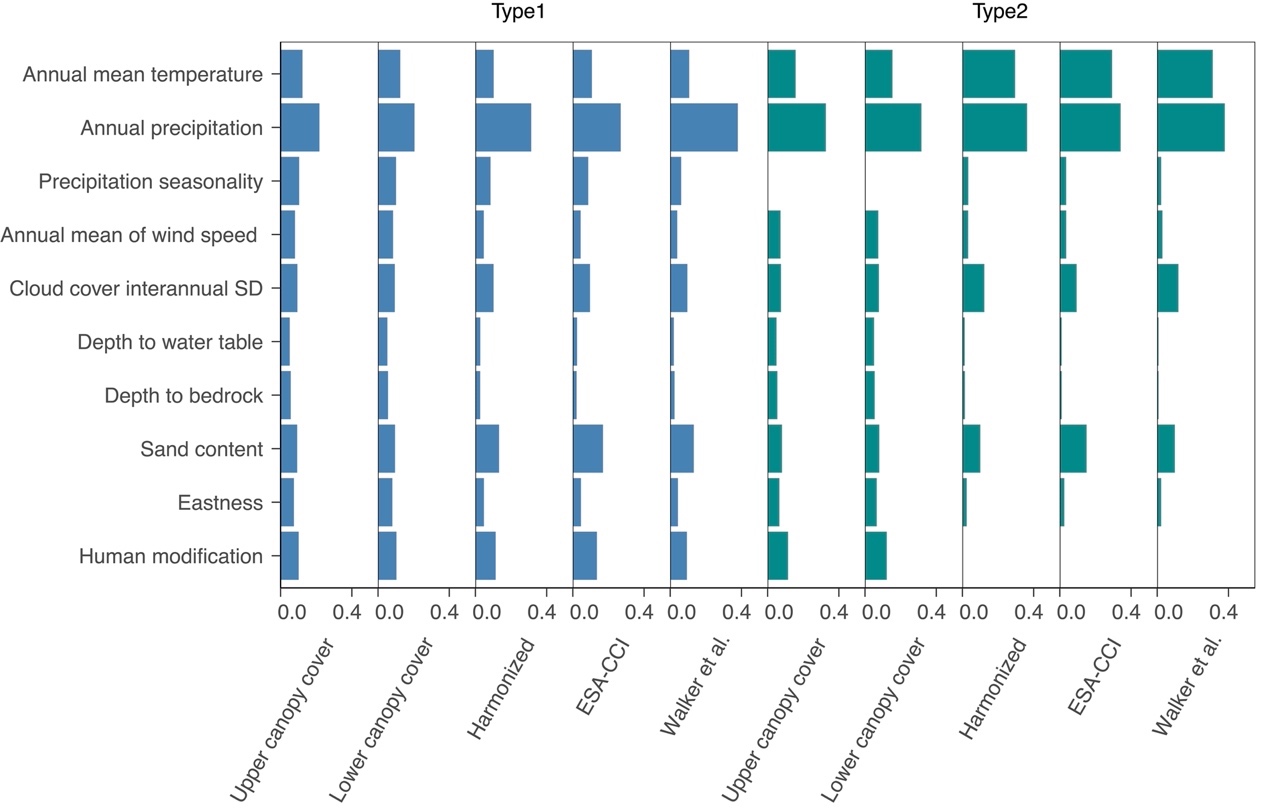


**Figure S14. Variable importance of 10 environmental metrics based on random forest MDA values for the ground-soured (*Upper/Lower canopy cover*) and satellite-derived (*Harmonized, ESA-CCI, Walker et al.*) models of tree carbon.** To represent the effects of climate, soil conditions, human disturbance and topography, we chose 10 variables out of the full set of 49 variables that show minimal collinearity among each other (VIF <5). The length of the bars represents the mean relative contribution of each variable to tree carbon density. The values were generated by calculating the mean of 20 bootstrapped random forest models.


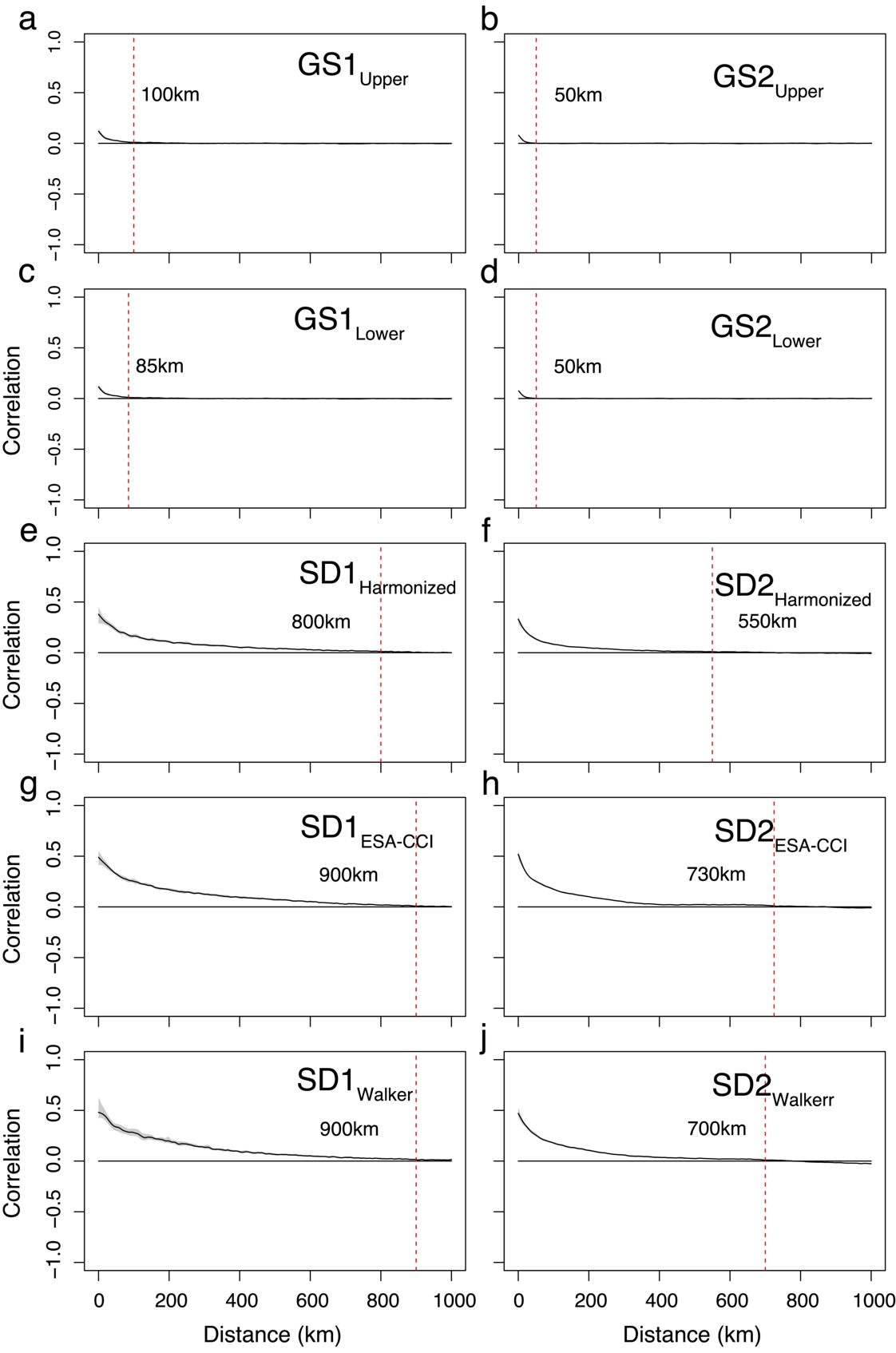


**Figure S15. Residual spatial autocorrelation (Moran’s I) of the ground-sourced (a-d) and satellite-derived (e-j) models, assessed using generalized additive models along a distance gradient.** Distances up to which there was positive spatial autocorrelation are shown as red dashed lines, and we applied these distances as buffer zone radii in the spatial leave-one-out cross validation. **a**, GS1_Upper_ model; **b**, GS2_Upper_ model; **c**, GS1_Lower_ model; **d**, GS2_Lower_ model; **e**, SD1_Harmonized_ model; **f**, SD2_Harmonized_ model; **g**, SD1_ESA-CCI_ model; **h**, SD2_ESA-CCI_ model; **i**, SD1_Walker_ model; and **j**, SD2_Walker_ model.

**Figure S16. Map showing the ratio between root and shoot biomass for global forest areas**. To account for tree carbon stored belowground as roots, we multiplied our aboveground tree carbon predictions by a spatially-explicit map of root-to-shoot ratio^12^.

**
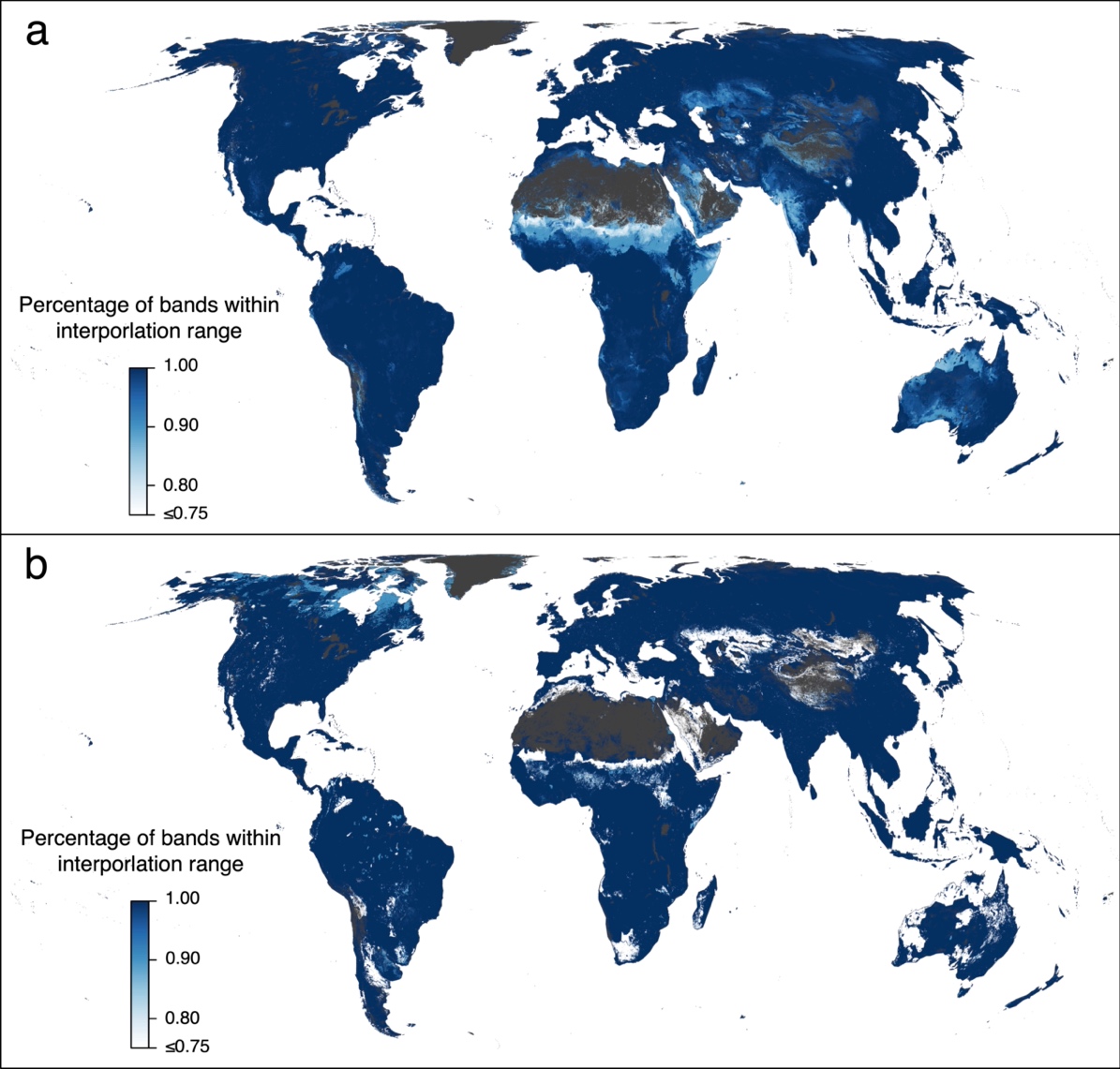
**

**Figure S17. Representation of the training data for the ground-sourced model considering all covariates (a) or only the human disturbance covariates (b).** To test for the extent of interpolation and extrapolation across all land pixels excluding Antarctica, we calculated the percentage of interpolation based on principal component analysis (PCA), that is, how often a pixel fell into the convex hull space of the bivariate combinations from the top principal components. **a**, To explore the representation of all covariates, we used 19 principal components with 171 combinations (see Methods). In total, 92% of the pixels fell within at least 95% of the PCA convex hulls. **B**, To explore the representation of the eight human disturbance covariates, we used 5 principal components with 10 combinations for eight human disturbance covariates (see Methods). In total, 90% of the pixels fell within at least 95% of the PCA convex hulls.

**Table S1. The global performance of the ground-sourced (SD) and satellite-derived (SD) models.** Coefficients of determination (R^2^ values) from 10-fold cross validation represent the means of the 100 best-performing models. The R^2^ values from spatially-buffered leave-one-out cross-validation (LOO-CV), accounting for the potential effect of spatial autocorrelation on model validation (Supplementary Figure S15), are shown in the right column.

| Model | Model name | R^2^ (10-fold CV) | R^2^ (LOO-CV) |
| --- | --- | --- | --- |
| GS | GS1_Upper canopy cover_ | 0.78 | 0.77 |
|  | GS2_Upper canopy cover_ | 0.75 | 0.74 |
|  | GS1_Lower canopy cover_ | 0.75 | 0.73 |
|  | GS2_Lower canopy cover_ | 0.72 | 0.70 |
| SD | SD1_Harmonized_ | 0.88 | 0.86 |
|  | SD2_Harmonized_ | 0.90 | 0.87 |
|  | SD1_ESA-CCI_ | 0.87 | 0.82 |
|  | SD2_ESA-CCI_ | 0.90 | 0.84 |
|  | SD1_Walker et al._ | 0.87 | 0.82 |
|  | SD2_Walker et al._ | 0.92 | 0.86 |

**Table S2. Live tree carbon potential within biomes.** Values represent total (below- and aboveground) live tree biomass in Gt carbon. Present: Total existing tree carbon. Total potential: Total carbon predicted across the entire potential tree coverage^6^ in the absence of humans. Difference: Difference between total potential and present carbon. Conservation: Share of the difference between total potential and present carbon stocks attributed to degradation of existing forest, i.e., the additional carbon that could be stored through conservation of existing forests. Restoration: Share of the difference between total potential and present carbon stocks attributed to deforestation, estimated as the tree carbon potential in regions that are currently not covered by trees. Values show the means of the four ground-sourced and six satellite-derived model predictions. Values in brackets show the full range across the ten models.

| Biome | Present | Total potential | Difference | Conservation | Restoration |
| --- | --- | --- | --- | --- | --- |
| Tropical moist | 206.5 (172.8-233.5) | 262.6 (220.6-299.5) | 56.1 (47.8-66.0) | 33.3 (28.7-38.5) | 22.8 (18.7-29.7) |
| Tropical dry | 7.3 (5.9-9.9) | 17.4 (13.4-23.0) | 10.1 (7.5-13.1) | 2.9 (2.1-4.4) | 7.2 (4.8-10.2) |
| Tropical coniferous | 2.7 (2.2-3.5) | 5.2 (4.0-6.6) | 2.4 (1.8-3.1) | 1.4 (0.8-2.0) | 1.0 (0.6-1.4) |
| Temperate broadleaf | 42.3 (32.9-49.2) | 69.1 (55.7-77.0) | 26.8 (22.8-27.7) | 9.6 (7.0-11.4) | 17.3 (12.3-20.1) |
| Temperate coniferous | 20.5 (14.9-24.4) | 26.8 (21.0-29.6) | 6.3 (6.1-5.2) | 3.7 (2.0-5.5) | 2.6 (1.8-2.8) |
| Boreal | 39.9 (21.2-57.7) | 49.7 (24.6-73.9) | 9.8 (3.4-16.1) | 7.0 (2.2-12.4) | 2.8 (1.2-4.1) |
| Tropical savanna | 43.5 (32.3-57.9) | 94.7 (66.0-122.2) | 51.2 (33.8-64.4) | 20.1 (12.6-36.5) | 31.0 (18.5-38.3) |
| Temperate savanna | 4.2 (2.5-5.4) | 20.6 (10.4-29.1) | 16.4 (7.9-23.7) | 1.1 (0.7-1.8) | 15.3 (7.2-22.2) |
| Flooded savanna | 1.8 (1.2-2.8) | 3.6 (2.3-5.0) | 1.8 (1.1-2.2) | 0.8 (0.5-1.3) | 1.0 (0.6-1.3) |
| Montane grassland | 3.4 (2.5-4.4) | 8.9 (5.4-12.8) | 5.5 (2.9-8.4) | 1.5 (0.9-2.1) | 4.1 (1.8-6.6) |
| Tundra | 4.0 (2.5-5.3) | 8.5 (3.8-13.7) | 4.5 (1.3-8.5) | 1.6 (0.2-3.4) | 2.9 (1.1-5.1) |
| Mediterranean forest | 3.1 (1.6-4.8) | 8.7 (4.3-11.7) | 5.6 (2.6-6.9) | 1.2 (0.7-1.8) | 4.4 (1.9-6.0) |
| Desert | 2.1 (1.4-2.7) | 21.1 (7.4-36.3) | 19.0 (6.0-33.6) | 1.1 (0.7-1.7) | 17.8 (5.3-32.2) |
| Mangroves | 1.8 (1.3-2.4) | 2.9 (2.4-3.5) | 1.0 (1.1-1.1) | 0.6 (0.4-0.8) | 0.4 (0.3-0.5) |
| Total | 383.1 (334.1-444.9) | 599.8 (487.1-711.7) | 216.7 (153.0-266.8) | 86.0 (72.0-107.5) | 130.7 (77.4-181.7) |

**Table S3. Live tree carbon stocks in the world’s forests.** Values represent below- and aboveground live tree carbon. See Table S2 for detailed description.

| Category | Present | Total potential | Difference | Conservation | Restoration |
| --- | --- | --- | --- | --- | --- |
| Tropical | 263.6 (225.5-304.8) | 386.3 (324.6-457.3) | 122.7 (99.2-152.5) | 59.2 (46.5-81.1) | 63.5 (52.6-80.2) |
| Temperate | 70.4 (52.8-82.8) | 125.5 (92.5-148.5) | 55.1 (39.7-65.6) | 15.9 (11.3-20.1) | 39.2 (23.1-52.1) |
| Boreal | 43.9 (23.7-63.0) | 58.2 (28.4-87.6) | 14.3 (4.8-24.6) | 8.6 (2.5-15.4) | 5.7 (2.2-9.2) |
| Dryland | 5.2 (3.0-7.5) | 29.8 (11.7-48.0) | 24.6 (8.6-40.5) | 2.3 (1.4-3.4) | 22.3 (7.2-38.1) |

**Table S4. Biome-level allometric equations of the non-tropical biomes from the GlobAllomeTree database**^8^ **and following the methodology of Jenkins et al**^9^**.** Equation: parameters of each allometric equation. R^2^: coefficient of determination of the DBH-based allometric models (Supplementary Figure S1).

| WWF Biome | Equation | R^2^ |
| --- | --- | --- |
| Temperate broadleaf | log(biomass) = -2.5021 + 2.5210 × log(DBH) | 0.98 |
| Temperate coniferous | log(biomass) = -2.3102 + 2.4560 × log(DBH) | 0.99 |
| Temperate savanna | log(biomass) = -1.9315 + 2.2659 × log(DBH) | 0.96 |
| Boreal | log(biomass) = -2.3322 + 2.3847 × log(DBH) | 0.98 |
| Desert | log(biomass) = -1.9653 + 2.2478 × log(DBH) | 0.99 |
| Mediterranean forest | log(biomass) = -2.3271 + 2.4102 × log(DBH) | 0.96 |
| Montane grassland | log(biomass) = -1.5386 + 2.1712 × log(DBH) | 0.93 |
| Tundra | log(biomass) = -2.4016 + 2.3494 × log(DBH) | 0.98 |

**Table S5. Biome-level carbon concentrations of woody biomass based on Martin et al.^13^.**

| Biome | Carbon concentration (%) |
| --- | --- |
| Tropical moist | 45.60 |
| Tropical dry | 45.70 |
| Tropical coniferous | 47.25 |
| Temperate broadleaf | 46.50 |
| Temperate coniferous | 50.10 |
| Boreal | 48.00 |
| Tropical savanna | 45.60 |
| Temperate savanna | 46.50 |
| Flooded savanna | 45.60 |
| Montane grassland | 46.50 |
| Tundra | 46.80 |
| Mediterranean forest | 47.75 |
| Desert | 47.75 |
| Mangroves | 45.60 |

**Table S6. List of the 49 covariates used in the models of carbon potential.**

| Variable | Type | Data resolution | Source and Reference |
| --- | --- | --- | --- |
| Annual mean temperature | Bioclimatic | ~1km | CHELSA Ver 1.2  [www.chelsa-climate.org](http://www.chelsa-climate.org)  Ref^14^ |
| Temperature annual range | Bioclimatic | ~1km |  |
| Temperature seasonality | Bioclimatic | ~1km |  |
| Isothermality | Bioclimatic | ~1km |  |
| Maximum temperature of warmest month | Bioclimatic | ~1km |  |
| Mean diurnal range | Bioclimatic | ~1km |  |
| Mean temperature of coldest quarter | Bioclimatic | ~1km |  |
| Mean temperature of driest quarter | Bioclimatic | ~1km |  |
| Mean temperature of warmest quarter | Bioclimatic | ~1km |  |
| Mean temperature of wettest quarter | Bioclimatic | ~1km |  |
| Minimum temperature of coldest month | Bioclimatic | ~1km |  |
| Precipitation seasonality | Bioclimatic | ~1km |  |
| Annual precipitation | Bioclimatic | ~1km |  |
| Precipitation of coldest quarter | Bioclimatic | ~1km |  |
| Precipitation of driest month | Bioclimatic | ~1km |  |
| Precipitation of driest quarter | Bioclimatic | ~1km |  |
| Precipitation of warmest quarter | Bioclimatic | ~1km |  |
| Precipitation of wettest month | Bioclimatic | ~1km |  |
| Precipitation of wettest quarter | Bioclimatic | ~1km |  |
| Solar radiation annual mean | Climatic | ~1km | WorldClim version 2.  <http://www.worldclim.com/version2>  Ref^15^ |
| Windspeed annual mean | Climatic | ~1km |  |
| Inter-annual standard deviation of cloud cover | Climatic | ~1km | EarthEnv  [www.earthenv.org/cloud](http://www.earthenv.org/cloud)  <http://www.earthenv.org/topography>  Ref^16,17^ |
| Intra-annual SD of cloud cover | Climatic | ~1km |  |
| Annual mean of cloud cover | Climatic | ~1km |  |
| Eastness | Topographic | ~1km |  |
| Elevation | Topographic | ~1km |  |
| Northness | Topographic | ~1km |  |
| Profile curvature | Topographic | ~1km |  |
| Roughness | Topographic | ~1km |  |
| Slope | Topographic | ~1km |  |
| Aspect cosine | Topographic | ~1km |  |
| Aspect sine | Topographic | ~1km |  |
| Aridity index | Soil | ~250m | Global Aridity Index and Potential Evapotranspiration (ET0) Climate Database version 2.0^18,19^ |
| Depth to water table | Soil | ~250m | SoilGrids  <https://soilgrids.org>  Ref^20^ |
| Absolute depth to bedrock | Soil | ~250m |  |
| Clay content 0-100cm | Soil | ~250m |  |
| Coarse fragments 0-100cm | Soil | ~250m |  |
| Sand content 0-100cm | Soil | ~250m |  |
| Silt content 0-100cm | Soil | ~250m |  |
| Soil pH H_2_O 0-100cm | Soil | ~250m |  |
| Cultivated and managed vegetation | Anthropogenic | ~1km | EarthEnv  <http://www.earthenv.org/landcover>  Ref^21^ |
| Urban builtup | Anthropogenic | ~1km |  |
| Human modification | Anthropogenic | ~1km | Kennedy et al.^22^ |
| Cropland | Anthropogenic | ~10km | HYDE 3.1  <https://www.pbl.nl/en/image/links/hyde>  Ref ^23,24^ |
| Grazing | Anthropogenic | ~10km |  |
| Pasture | Anthropogenic | ~10km |  |
| Rangeland | Anthropogenic | ~10km |  |
| Protected area | Anthropogenic | Polygon | World Database on Protected AreasRef^25,26^ |
| Present tree cover | Vegetative | ~30m | Hansen et al., ^27^ |
| Potential tree cover | Vegetative | ~1km | Bastin et al., ^6^ |

**Table S7. Estimates of the global living tree carbon potential from previous studies.** The “Full” estimates represent the total carbon that could be stored in global forests in the absence of human activities (Figure 3b). The “Difference” estimates represent the additional potential carbon that could be stored in the global forests, i.e., the difference between potential and present carbon stocks (Figure 3d). These estimates were derived from four different approaches (“Model type”): inventory-based empirical estimates, mechanistic models, ensemble models, and data-driven models (see Methods).

| Model type | Gt carbon | Type | References |
| --- | --- | --- | --- |
| Ensemble | 1089.8 | Full | Ref^28–31^. |
| Inventory | 980.0 | Full | Ref^32^. |
| Ensemble | 977.6 | Full | Ref^33–35^ |
| Inventory | 971.0 | Full | Ref.^29^ |
| Inventory | 956.0 | Full | Ref.^36^ |
| Inventory | 924.0 | Full | Ref.^37^ |
| Mechanistic model | 923.0 | Full | Ref.^38^ |
| Ensemble | 916.0 | Full | Ref.^28^ |
| Inventory | 907.6 | Full | Ref.^5,39^ |
| Inventory | 900.0 | Full | Ref.^31^ |
| Inventory | 877.5 | Full | Ref.^5,40^ |
| Mechanistic model | 857.0 | Full | Ref.^41^ |
| Mechanistic model | 850.0 | Full | Ref.^42^ |
| Data-driven models | 795.5 | Full | Ref.^1^ |
| Mechanistic model | 772.0 | Full | Ref.^43^ |
| Ensemble | 771.0 | Full | Ref^28–31^. |
| Inventory | 737.0 | Full | Ref.^44^ |
| Mechanistic model | 695.0 | Full | Ref.^45^ |
| Mechanistic model | 641.0 | Full | Ref.^46^ |
| Ensemble | 610.0 | Full | Ref.^47^ |
| Mean ± SD | **857.5 ± 123.7** | **Full** |  |
| Ensemble | 466.0 | Difference | Ref.^28^ |
| Inventory | 378.6 | Difference | Ref.^48^ |
| Data-driven models | 354.4 | Difference | Ref.^1^ |
| Inventory | 340.0 | Difference | Ref.^31^ |
| Inventory | 319.0 | Difference | Ref.^29^ |
| Mechanistic model | 254.0 | Difference | Ref.^45^ |
| Inventory | 242.0 | Difference | Ref.^44^ |
| Mechanistic model | 183.8 | Difference | Ref.^42^ |
| Mechanistic model | 150.0 | Difference | Ref.^42^ |
| Mean ± SD | **298.6 ± 100.1** | **Difference** |  |

**References**

1. Walker, W. *et al.* The global potential for increased storage of carbon on land. *Proc. Natl. Acad. Sci.* 1–12 (2022). doi:10.1073/pnas.2111312119

2. Santoro, M. & Cartus, O. *ESA Biomass Climate Change Initiative (Biomass_cci): Global datasets of forest above-ground biomass for the years 2010, 2017 and 2018, v2*. (2021). doi:10.5285/84403d09cef3485883158f4df2989b0c.

3. Santoro, M. *et al.* *GlobBiomass Global Above-Ground Biomass and Growing Stock Volume Datasets, available on-line at http://globbiomass.org/products/global-mapping*. (2018).

4. Spawn, S. A., Sullivan, C. C., Lark, T. J. & Gibbs, H. K. Harmonized global maps of above and belowground biomass carbon density in the year 2010 Authors Background & Summary. *Sci. Data* **7**, 112 (2020).

5. Ruesch, a. S. & Gibbs, H. H. K. *New Global Biomass Carbon Map for the Year 2000 Based On IPCC Tier-1 Methodology*. *Carbon Dioxide Information Analysis Center, Oak Ridge National Laboratory, Oak Ridge, USA* (2008).

6. Bastin, J. F. *et al.* The global tree restoration potential. *Science* **364**, 76–79 (2019).

7. Sanderman, J., Hengl, T. & Fiske, G. J. Soil carbon debt of 12,000 years of human land use. *Proc. Natl. Acad. Sci.* **114**, 9575–9580 (2017).

8. Henry, M. *et al.* GlobAllomeTree: international platform for tree allometric equations to support volume, biomass and carbon assessment. *Iforest* **6**, 326–330 (2013).

9. Jenkins, J. C., Chojnacky, D. C., Heath, L. S. & Birdsey, R. A. National-scale biomass estimators for United States tree species. *For. Sci.* **49**, 12–35 (2003).

10. Chave, J. *et al.* Improved allometric models to estimate the aboveground biomass of tropical trees. *Glob. Chang. Biol.* **20**, 3177–3190 (2014).

11. Réjou-Méchain, M., Tanguy, A., Piponiot, C., Chave, J. & Hérault, B. biomass: an r package for estimating above-ground biomass and its uncertainty in tropical forests. *Methods Ecol. Evol.* **8**, 1163–1167 (2017).

12. Ma, H. *et al.* The global distribution and environmental drivers of aboveground versus belowground plant biomass. *Nat. Ecol. Evol.* 1–13 (2021).

13. Martin, A. R., Doraisami, M. & Thomas, S. C. Global patterns in wood carbon concentration across the world’s trees and forests. *Nat. Geosci.* **11**, 915–920 (2018).

14. Karger, D. N. *et al.* Climatologies at high resolution for the earth’s land surface areas. *Sci. data* **4**, 170122 (2017).

15. Fick, S. E. & Hijmans, R. J. WorldClim 2: new 1-km spatial resolution climate surfaces for global land areas. *Int. J. Climatol.* **37**, 4302–4315 (2017).

16. Wilson, A. M. & Jetz, W. Remotely sensed high-resolution global cloud dynamics for predicting ecosystem and biodiversity distributions. *PLoS Biol.* **14**, e1002415 (2016).

17. Amatulli, G. *et al.* A suite of global, cross-scale topographic variables for environmental and biodiversity modeling. *Sci. data* **5**, 180040 (2018).

18. Trabucco, A. & Zomer, R. J. Global Soil Water Balance Geospatial Database. CGIAR Consortium for Spatial Information. In, Published online, available from the CGIAR-CSI GeoPortal. *www. cgiar-csi. org* (2010).

19. Zomer, R. J., Trabucco, A., Bossio, D. A. & Verchot, L. V. Climate change mitigation: A spatial analysis of global land suitability for clean development mechanism afforestation and reforestation. *Agric. Ecosyst. Environ.* **126**, 67–80 (2008).

20. Shangguan, W., Hengl, T., de Jesus, J. M., Yuan, H. & Dai, Y. Mapping the global depth to bedrock for land surface modeling. *J. Adv. Model. Earth Syst.* **9**, 65–88 (2017).

21. Tuanmu, M.-N. & Jetz, W. A global, remote sensing-based characterization of terrestrial habitat heterogeneity for biodiversity and ecosystem modelling. *Glob. Ecol. Biogeogr.* **24**, 1329–1339 (2015).

22. Kennedy, C. M., Oakleaf, J. R., Theobald, D. M., Baruch-Mordo, S. & Kiesecker, J. Managing the middle: A shift in conservation priorities based on the global human modification gradient. *Glob. Chang. Biol.* **25**, 811–826 (2019).

23. Klein Goldewijk, K., Beusen, A. & Janssen, P. Long-term dynamic modeling of global population and built-up area in a spatially explicit way: HYDE 3.1. *The Holocene* **20**, 565–573 (2010).

24. Klein Goldewijk, K., Beusen, A., Van Drecht, G. & De Vos, M. The HYDE 3.1 spatially explicit database of human-induced global land-use change over the past 12,000 years. *Glob. Ecol. Biogeogr.* **20**, 73–86 (2011).

25. UNESCO. The World Database on Protected Areas. (2011).

26. UNEP-WCMC, I. Protected Planet: the World Database on Protected Areas (WDPA). *UNEP-WCMC IUCN, Cambridge, UK Available http//www. Prot. net, Accessed date* **21**, (2018).

27. Hansen, M. C. *et al.* High-Resolution Global Maps of 21st-Century Forest Cover Change. *Science* **342**, 850–853 (2013).

28. Erb, K.-H. *et al.* Unexpectedly large impact of forest management and grazing on global vegetation biomass. *Nature* **553**, 73 (2018).

29. Roy, J., Mooney, H. A. & Saugier, B. *Terrestrial global productivity*. (Elsevier, 2001).

30. Olson, D. M. *et al.* Terrestrial Ecoregions of the World: A New Map of Life on EarthA new global map of terrestrial ecoregions provides an innovative tool for conserving biodiversity. *Bioscience* **51**, 933–938 (2001).

31. Ajtay, G. L. Terrestrial primary production and phytomass. *Glob. carbon cycle, SCOPE 13* 129–181 (1979).

32. Bazilevich, N. I., Rodin, L. Y. & Rozov, N. N. Geographical aspects of biological productivity. *Sov. Geogr.* **12**, 293–317 (1971).

33. Saatchi, S. S. *et al.* Benchmark map of forest carbon stocks in tropical regions across three continents. *Proc. Natl. Acad. Sci.* **108**, 9899–9904 (2011).

34. Baccini, A. *et al.* Estimated carbon dioxide emissions from tropical deforestation improved by carbon-density maps. *Nat. Clim. Chang.* **2**, 182 (2012).

35. Thurner, M. *et al.* Carbon stock and density of northern boreal and temperate forests. *Glob. Ecol. Biogeogr.* **23**, 297–310 (2014).

36. Olson, J. S., Watts, J. A. & Allison, L. J. *Carbon in live vegetation of major world ecosystems*. (Oak Ridge National Laboratory, 1983).

37. Adams, J. M., Faure, H., Faure-Denard, L., McGlade, J. M. & Woodward, F. I. Increases in terrestrial carbon storage from the Last Glacial Maximum to the present. *Nature* **348**, 711–714 (1990).

38. Sitch, S. *et al.* Evaluation of ecosystem dynamics, plant geography and terrestrial carbon cycling in the LPJ dynamic global vegetation model. *Glob. Chang. Biol.* **9**, 161–185 (2003).

39. Pan, Y. *et al.* A large and persistent carbon sink in the world’s forests. *Science* **333**, 988–993 (2011).

40. FAO. Global Forest Resources Assessment 2010. (2010).

41. Kaplan, J. O. *et al.* Holocene carbon emissions as a result of anthropogenic land cover change. *The Holocene* **21**, 775–791 (2011).

42. Shevliakova, E. *et al.* Carbon cycling under 300 years of land use change: Importance of the secondary vegetation sink. *Global Biogeochem. Cycles* **23**, (2009).

43. Prentice, I. C., Harrison, S. P. & Bartlein, P. J. Global vegetation and terrestrial carbon cycle changes after the last ice age. *New Phytol.* **189**, 988–998 (2011).

44. West, P. C. *et al.* Trading carbon for food: Global comparison of carbon stocks vs. crop yields on agricultural land. *Proc. Natl. Acad. Sci.* **107**, 19645–19648 (2010).

45. Hurtt, G. C. *et al.* Harmonization of land-use scenarios for the period 1500--2100: 600 years of global gridded annual land-use transitions, wood harvest, and resulting secondary lands. *Clim. Change* **109**, 117 (2011).

46. Krinner, G. *et al.* A dynamic global vegetation model for studies of the coupled atmosphere‐biosphere system. *Global Biogeochem. Cycles* **19**, (2005).

47. Siegenthaler, U. & Sarmiento, J. L. Atmospheric carbon dioxide and the ocean. *Nature* **365**, 119 (1993).

48. Pan, Y., Birdsey, R. A., Phillips, O. L. & Jackson, R. B. The structure, distribution, and biomass of the world’s forests. *Annu. Rev. Ecol. Evol. Syst.* **44**, 593–622 (2013).
